# The ‘analysis of gene expression and biomarkers for point-of-care decision support in Sepsis‘ study; temporal clinical parameter analysis and validation of early diagnostic biomarker signatures for severe inflammation andsepsis-SIRS discrimination

**DOI:** 10.3389/fimmu.2023.1308530

**Published:** 2024-01-25

**Authors:** Tamas Szakmany, Eleanor Fitzgerald, Harriet N. Garlant, Tony Whitehouse, Tamas Molnar, Sanjoy Shah, Dong Ling Tong, Judith E. Hall, Graham R. Ball, Karen E. Kempsell

**Affiliations:** ^1^ Department of Anaesthesia, Intensive Care and Pain Medicine, Division of Population Medicine, Cardiff University, Cardiff, United Kingdom; ^2^ Anaesthesia, Critical Care and Theatres Directorate, Cwm Taf Morgannwg University Health Board, Royal Glamorgan Hospital, Llantrisant, United Kingdom; ^3^ UK Health Security Agency, Porton Down, Salisbury, United Kingdom; ^4^ NIHR Surgical Reconstruction and Microbiology Research Centre, Queen Elizabeth Hospital, Mindelsohn Way Edgbaston, Birmingham, United Kingdom; ^5^ Critical Care Directorate, University Hospitals Bristol and Weston NHS Foundation Trust, Bristol, United Kingdom; ^6^ Faculty of Information and Communication Technology, Universiti Tunku Abdul Rahman, Kampar, Perak, Malaysia; ^7^ Medical Technology Research Facility, Anglia Ruskin University, Essex, United Kingdom

**Keywords:** sepsis, biomarker, SIRS, diagnostic, severe inflammation, mRNA signature

## Abstract

**Introduction:**

Early diagnosis of sepsis and discrimination from SIRS is crucial for clinicians to provide appropriate care, management and treatment to critically ill patients. We describe identification of mRNA biomarkers from peripheral blood leukocytes, able to identify severe, systemic inflammation (irrespective of origin) and differentiate Sepsis from SIRS, in adult patients within a multi-center clinical study.

**Methods:**

Participants were recruited in Intensive Care Units (ICUs) from multiple UK hospitals, including fifty-nine patients with abdominal sepsis, eighty-four patients with pulmonary sepsis, forty-two SIRS patients with Out-of-Hospital Cardiac Arrest (OOHCA), sampled at four time points, in addition to thirty healthy control donors. Multiple clinical parameters were measured, including SOFA score, with many differences observed between SIRS and sepsis groups. Differential gene expression analyses were performed using microarray hybridization and data analyzed using a combination of parametric and non-parametric statistical tools.

**Results:**

Nineteen high-performance, differentially expressed mRNA biomarkers were identified between control and combined SIRS/Sepsis groups (FC>20.0, p<0.05), termed ‘indicators of inflammation’ (I°I), including CD177, FAM20A and OLAH. Best-performing minimal signatures e.g. FAM20A/OLAH showed good accuracy for determination of severe, systemic inflammation (AUC>0.99). Twenty entities, termed ‘SIRS or Sepsis’ (S°S) biomarkers, were differentially expressed between sepsis and SIRS (FC>2·0, p-value<0.05).

**Discussion:**

The best performing signature for discriminating sepsis from SIRS was CMTM5/CETP/PLA2G7/MIA/MPP3 (AUC=0.9758). The I°I and S°S signatures performed variably in other independent gene expression datasets, this may be due to technical variation in the study/assay platform.

## 1 Introduction

Sepsis is a major contributor to avoidable deaths worldwide and is considered one of the most common causes of hospital admission and inpatient deterioration (1). In 2017, eleven million sepsis related deaths were estimated globally, equivalent to one in every five deaths being sepsis associated (2). In the UK, at least 200,000 episodes of sepsis are now predicted annually with around 48,000 associated deaths at an estimated cost of £1·5-2 billion each year to the NHS and £11 billion to the wider economy (1). A key challenge in diagnosis and management of sepsis is early recognition (3). Additional complications of diagnosing sepsis are distinguishing between this and patients with Systemic Inflammatory Response Syndrome of non-infectious origin (SIRS) e.g., trauma, surgery, thrombosis, ‘out of hospital cardiac arrest’ (OOHCA) etc., as many of its clinical signs and symptoms are highly similar (3–5). Current diagnostic methods struggle to differentiate between sepsis and other conditions, exacerbated by the difficulties of obtaining microbiological culture results from localized acute infections (6).

The current definition of sepsis describes the condition as a ‘dysregulated host response to infection leading to organ dysfunction; where an inappropriate inflammatory response causes significant damage to itself in an attempt to resolve infection’, with the addition of organ dysfunction being the latest update to clinical definition (4). Since the trajectory of the systemic immuno-inflammatory response in sepsis can alternate between hyper-activity and immunosuppression, any uncorrected, escalating deviation from homeostasis in either direction can result in a high risk of secondary infections, multi-organ failure and death (7–9). Hyper-activation and suppression of the immune system are both anticipated to be occurring at the same time, therefore, understanding the underlying pathology and providing effective diagnosis and treatment regimens remains a significant challenge (10).

Whole blood transcriptomics have been used to facilitate understanding of this diverse sepsis immune response and to identify potential targets for diagnosis and treatment (11–13). Many of these studies used total RNA isolated from PBLs, and high throughput quantification of gene expression levels. These methods have been successfully used in other diseases e.g., cancer, trauma, infections etc. to identify clinically relevant subgroups with potentially distinct treatment responses (12–14). These studies show promise, as panels of biologically relevant biomarkers which can reliably, accurately, and quickly distinguish sepsis from other conditions have been identified, particularly non-infection induced SIRS and further categorize sepsis based on the source of the infection, abdominal (ABDM), pulmonary (PLMN) etc. Numerous sepsis diagnostic signatures have now been published including Septicyte Lab, Sepsis Meta Score/InSep (Inflammatix) (13, 15–23). The performance of these signatures has been evaluated and shown to be reasonable but inconsistent between studies (17, 24). Researchers are still seeking a combination of biomarkers which must be highly specific and sensitive and detectable using minimally invasive sampling procedures.

We have developed a bioinformatics framework for meta-analysis of previously published datasets and identified key hub and/or associated biomarkers, which show potential for diagnostic use in identification of severe inflammation and discrimination of SIRS from Sepsis (25). Here we describe a prospective clinical validation study to further characterize these biomarker signatures for (i) severe inflammation termed ‘Indicators of Inflammation’ ((I°I) upregulated in both SIRS and sepsis compared to controls) and (ii) ‘SIRS or Sepsis’ ((S°S) differentially upregulated in either SIRS or sepsis), in a cohort of newly recruited patients with ABDM or PLMN sepsis and a SIRS group consisting of patients admitted following OOHCA, in comparison to healthy controls.

## 2 Materials and methods

### 2.1 Study design

We performed a prospective, observational study, where eligible patients were consecutively recruited. Given the observational nature of our study, patients were treated according to local best practice guidelines in the respective ICUs.

### 2.2 Ethical approval

The Analysis of geNe Expression and bioMarkers fOr poiNt-of-care dEcision support in Sepsis (ANEMONES) study was approved by the South Wales Research Ethics Committee Panel D; Ref: 12/WA/0303 and retrospectively registered at ISRCTN99754654. Participants, or if incapacitated, their relatives or their professional legal representatives provided written informed consent.

### 2.3 Patient recruitment, blood sampling and processing

Consecutive patients meeting specified criteria of severe sepsis due to infection in the pulmonary (PLMN) or abdominal (ABDM) systems - together termed as sepsis, or severe inflammation causing organ dysfunction with no clinical suspicion of infection following OOHCA – termed as SIRS, were recruited from four UK hospitals (Royal Glamorgan Hospital, Prince Charles Hospital, Bristol Royal Infirmary and University Hospitals Birmingham) between 2013 and 2015. Healthy control blood samples were collected from volunteers once at the Day1 timepoint only, at The UK Health Security Agency (UKHSA), Porton Down (n=30). Detailed inclusion and exclusion criteria for all groups is provided in the **Supplementary Material File S1**. We intended to recruit 160 patients with severe sepsis and septic shock as defined by the 2001 Sepsis 2.0 definition and 40 patients with SIRS and organ failure not related to infection. Detailed inclusion and exclusion criteria are provided in the Supplement. No published data existed at the time of study inception to justify a formal power calculation; the sample size was based on a compromise between desirability and achievability. Due to our strict inclusion criteria, all sepsis patients met the updated Sepsis 3.0 criteria, published following the completion of our recruitment in 2016 (3).

Blood samples were collected from sepsis and SIRS patients at Day1, Day2 and Day5 of admittance to an intensive care unit (ICU) and on discharge. Some timepoints were not collected due to patient death, patients leaving ICU or events beyond our control. Healthy control blood samples were collected from volunteers once at the Day1 timepoint only. On collection, 5ml of whole heparinized blood was mixed with Erythrocyte Lysis (EL) Buffer (QIAGEN) followed by incubation for 10-15 minutes at room temperature. Peripheral blood leukocytes (PBLs) were recovered from erythrocyte lysed blood by centrifugation at 400 x g for 10 minutes at 4˚C and resuspended in a further 2ml of EL buffer. PBLs were recovered again by centrifugation at 400 x g for 10 minutes at 4˚C and stored at -80°C prior to ongoing analysis.

### 2.4 Statistical analysis of clinical parameters

Clinical parameters including laboratory values etc. were assessed for normality across the sample groups prior to ongoing statistical analyses using GraphPad Prism 9·0 (GPP9). (**Supplementary Information file (SIf) S1**, **Supplementary Table S1·4**). As variables showed a predominantly non-normal distribution, all statistical analyses were conducted using Mann-Whitney U tests using GPP9 (**Supplementary Information file (SIf) S1**, **Supplementary Tables S1·5** to **S1·8**; **Supplementary Table S1·5** (SIRS vs sepsis (PLMN and ABDM combined)), **Supplementary Table S1·6** (SIRS vs ABDM) **Supplementary Table S1·7** (SIRS vs PLMN), **Supplementary Table S1·8** (PLMN vs ABDM) and summarized in **Supplementary Table S1·9**). Graphical outputs were depicted using median boxplot, correlation coefficient maps and other functions in GPP9 and Sigmaplot 12·0 (SP12·0).

### 2.5 mRNA purification and microarray hybridisation

RNA was prepared from patient PBLs using a semi-automated process using the Maxwell^®^ 16 platform and the Maxwell^®^ 16 LEV simplyRNA Blood Kit. Concentration and purity (A280/260 ratio ≥ 1·8) were assessed by spectrophotometry using a Nanodrop ND-1000 Spectrophotometer (Thermo Scientific). mRNA purified from PBLs was labeled with Cy3 using the Agilent QuickAmp one color labeling kit and then hybridized to Human SurePrint G3 Human Gene Expression v2 8x60K Microarrays according to the manufacturer’s instructions. After hybridization and wash steps, the slides were scanned using an Agilent Surescan Dx G5761AA Microarray Scanner using default settings. All annotations, normalized and raw data are deposited in GEO under accession number GSE236713 at the National Center for Biotechnology Information (NCBI), National Library of Medicine, National Institutes of Health (NIH), United States of America.

### 2.6 Preparation of microarray data

Raw numeric values were exported from the Agilent Surescan Dx G5761AA Microarray Scanner and uploaded into GeneSpring 14·9 (GX14.9) bioinformatics software for processing. All imported, raw data were normalized to the 75^th^ percentile and baseline transformed using the global median prior to further analysis. These were sorted into disease relevant groups i.e., healthy controls (CNTRL), SIRS, ABDM and PLMN sepsis and further stratified by day of sample i.e., Day1 2, 5 and discharge, clinical outcome i.e., did not survive (DNS)/survived (S). Normalized data were further analyzed using combinations of these group categorizations using GX14·9, receiver operating characteristics (ROC) curve analysis and random forest (RF) modeling scripts run in either in ‘R’, SigmaPlot 14·9 or GraphPad Prism 9·0 and artificial neural networks (ANN).

### 2.7 Statistical analyses: GeneSpring™ 14·9

Normalized data were further analyzed using various statistical packages and other functions in GX14·9. Data were assessed for quality (50739 total entities) and were then filtered by expression (between values -7·0 to 7·0) to remove outliers (50728 remaining entities, six predominantly X and Y-chromosome linked genes were removed DDX3Y, PSPHP1, XIST, RPS4Y1, RPS4Y2 and BTNL8). Statistically significant features were identified using either one-way ANOVA, Principal Component Analysis (PCA) or T-test analyses, using the Benjamin-Hochberg False Discovery Rate (BH-FDR) multiple testing correction at a cut-off of p <0.05. Fold change cut-off analyses were conducted using a default cut-off setting of >2.0. Data were further processed and depicted graphically using Euclidian hierarchical cluster analysis, heatmaps and other GX14.9 functions using default settings.

### 2.8 Statistical analyses: artificial neural networks

Normalized data were also analyzed using a stepwise Artificial Neural Network (ANN) approach incorporating Monte Carlo cross validation and a supervised learning approach, applied to a three-layer multilayer perception architecture. This was used to identify an optimized gene signature panel comprising orthogonal genes from a previously established gene biomarker set for sepsis. The stepwise ANN model comprised of 3-layer architecture and backpropagation learning with embedded exhaustive search strategy and cross-validation procedure. The approach was repeated five to ten times in stepwise additions, to assess the stability of the identified gene set given the number of cases provided. This was achieved using a stochastics data selection approach incorporating Monte Carlo cross-validation. The ANN modeling undertaken used a supervised learning approach applied to three-layered multi-layer perception architecture. The initial weight matrix was randomized with a standard deviation of 0.1 to reduce the risk of over-fitting the data. The ANN architecture was initially constrained to two hidden nodes in the hidden layer also for this reason. Hidden nodes and the output node incorporated a sigmoidal transfer function. During training weights were updated by a feedforward backpropagation algorithm (26). Learning rate and momentum were set at 0.1 and 0.5, respectively. The output node was coded as 0 if the patient showed no evidence of sepsis and 1.0 if sepsis was evident. Similar assessments were performed for patients with SIRS.

Prior to ANN training, the data was randomly divided into three subsets; 60% for training, 20% for testing (to assess model performance during the training process) and 20% for Monte Carlo cross-validation (to independently test the model on data completely blind to the model). This process of random sample cross-validation also contributed to the reduction of over-fitting to the data and assess how well the model would perform on a blind data set. The normalized intensity of each gene was used an individual input in the ANN model, creating *n* individual models, where *n* was the number of genes in the provided panel. These *n* models were then split into three subsets (as described above) and trained. This random resampling and training process was repeated 50 times to generate predictions and associated error values for each sample with respect to the validation (blind) data. Imputes were ranked in ascending order based on predictive error and the gene that performed with the lowest error was selected for further training. Next, each of the remaining genes were sequentially added to the previous best gene, and were used in combination in a model, creating *n-1* models each containing two gene inputs. Training was repeated and performance evaluated. The model with the highest modeling performance was again selected and the process repeated creating *n-2* models each containing three inputs. This resulted in a final model containing the expression signature that most accurately classified the patients according to severe inflammation, SIRS or sepsis or other investigative interrogations.

### 2.9 Random forest modeling and biomarker selection

Random forest (RF) modeling (27) was performed using the ‘RandomForest’ package in 'R' programming to identify biomarkers of most importance from both I°I and S°S biomarker panels and identify best candidates for use in diagnostic signatures. Classification models were performed on each of the I°I biomarkers and S°S biomarkers panels using normalized Day1 data randomly split (75% training cohort and 25% testing cohort). For biomarker selection, variables were ranked on decrease in accuracy and Gini scores. The Gini score indicated how often a random sample from the test set would be incorrectly categorized as having good or poor prognosis if the samples were randomly distributed (27–29).

### 2.10 Receiver operating characteristic curve analyses

Receiver operating characteristic curve (ROC) analyses were performed on biomarkers identified as most important individually using the ‘ROCR’ package in 'R', the ROC analysis tools in SigmaPlot 12.0 or GraphPad Prism 9.0. Selected biomarkers were then combined additively into diagnostic signatures to produce a composite panel score on which ROC analysis was performed to identify best performing combinations. Best performing signatures were identified based not only on their Area under the ROC (AUC) value and 90% CI as a measure of accuracy but on their Positive and Negative predictive values (PPV/NPV) at various cut-offs. Best cut-off values were predicted by measuring the optimal accuracy of the curve, from which sensitivity and specificity values were calculated.

### 2.11 Evaluation of biomarker signatures using other previously published datasets

Four candidate S°S signatures and two candidate I°I signatures were evaluated on previously published (microarray, PCR, RNAseq) Sepsis datasets from Herwanto et al. (30) (GSE154918), Martinez-Paz et al. (31) (GSE131761), Tang et al. (32)(GSE9960), Sutherland et al. (33) (GSE28750), Scicluna et al. (22), (GSE65682). These datasets were selected under the following criteria: i) adult patients (as opposed to pediatric), ii) data availability for all biomarkers of interest, iii) must contain appropriate groups e.g., control, SIRS, Sepsis. A COVID-19 dataset containing a bacterial infection sample group from McClain et al (34) GSE161731 was also included for evaluation of I°I biomarkers to determine if these biomarkers were specific to severe systemic inflammation and/or sepsis. Processed data was extracted for all biomarkers of interest and ROC analysis performed on composite panel scores generated from these data as previously described.

## 3 Results

### 3.1 Clinical study overview

**Figure 1:**
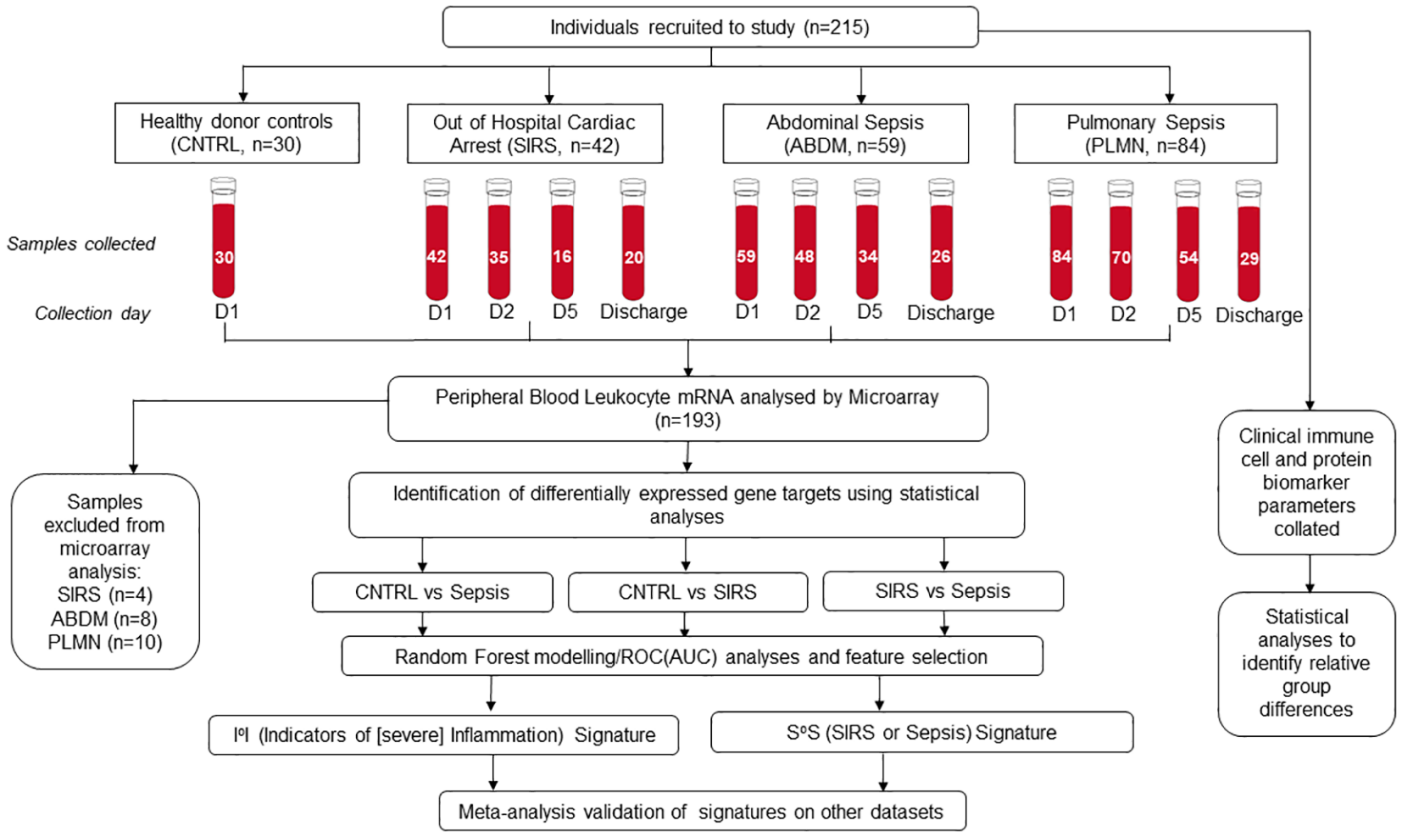
Schematic overview of clinical study, recruitment, sample collection and processing, microarray hybridization and data analysis.

Fifty-nine patients with ABDM, eighty-four patients with PLMN and forty-two patients with SIRS (OOHCA) were recruited over the study period. Thirty healthy volunteers were also enrolled as controls (CNTRL) (**Figure 1** - study overview). Patients were excluded from the analysis if insufficient patient information was available at the time of laboratory arrival, or if no samples were collected across timepoints. Demographic, clinical-scoring assessment, and immune cellular information are summarized in **Table 1** with detailed information including inclusion and exclusion criteria, cellular, microbiological and short-term prognosis information in **Supplementary Information file (SIf) S1**, **Supplementary Table 1.1** to **Supplementary Table 1.3**; **Supplementary Table 1.1** (SIRS), **Supplementary Table 1.2** (Sepsis; PLMN and ABDM), **Supplementary Table 1.3** (CNTRL). No clinical, cellular or microbiological information was collected for the CNTRL group, with samples collected for the Day1 timepoint only. The ABDM, PLMN and SIRS groups were well matched for age, however the CNTRL group was almost 20 years younger (**Table 1**). There was a sex bias in the CNTRL and SIRS groups; 70% female in the CNTRL group and 81% male in the SIRS group.

**Table 1:** Demographic and group information for clinical study samples for Sex (Male/Female), age, sex unknown and survival (%) APACHE score (Day1 only) and SOFA score Days 1,2, 5 and discharge.

	Healthy Donor Controls (CNTRL)	Severe inflammatory Response Syndrome (SIRS (OOHCA))	Abdominal Sepsis (ABDM)	Pulmonary Sepsis (PLMN)	Combined Sepsis (ABDM+PLMN)	Statistical Significance		
						SIRS vs ABDM	SIRS vs PULM	SIRS vs Combined Sepsis
**Male (n)**	9	34	27	45	72			
**Female (n)**	21	8	30	39	69			
**Age**	41 (33-47.75)	62 (50.25-70.75)	72 (63.25-77)	67 (52.5-77)	68 (59-76)			
**Sex unknown (n)**	0	0	2	0	2			
**Survival (%)**	100	61.11	74.51	78.38	76.44			
**Apache II score D1**	–	30 (27-34)	31 (24-37)	31 (23-40)	31 (22-38)			
**SOFA score D1**	–	16 (14-20)	17 (14-19)	17 (15-19)	17 (14-19)			
**SOFA score D2**	–	17 (15-18)	17 (14-19)	16 (14-18)	17 (14-18)			
**SOFA Score D5**	–	14 (11-15)	14 (11-18)	14 (12-16)	14 (11-17)			
**SOFA score Discharge**	–	9 (9-11)	8 (7-9)	9 (7-10)	8 (7-10)			
**CRP D1**	-	26 (5-51)	216 (104-336)	197 (117-280)	202 (108-300)	<0.0001	<0.0001	<0.0001
**WBC D1**	–	14.9 (9.3-21.2)	15.8 (11.7-19.4)	15.8 (10.2-22.4)	16.0 (10.7-21.3)			
**Neutrophils D1**	–	12.4 (7.3-18.8)	12.8 (9.6-17.3)	13.4 (8.6-20.2)	13.4 (8.7-18.3)			
**Lymphocytes D1**	-	1.0 (0.6-1.5)	0.7 (0.5-1.1)	0.6 (0.4-1.0)	0.7 (0.4-1.0)	0.0045	0.0002	0.0002
**Basophils D1**	-	0.01 (0.00-0.02)	0.01 (0.00-0.01)	0.01 (0.00-0.02)	0.01 (0.00-0.10)			0.0436
**Platelets D1**	–	191 (158-234)	205 (133-301)	206 (143-284)	206 (135-296)			
**CRP D2**	-	131 (68-170)	251 (113-339)	201 (116-273)	208 (115-295)	<0.0001	0.0010	<0.0001
**WBC D2**	-	10.4 (7.7-15.5)	14.8 (9.8-18.7)	15.1 (10.2-19.6)	14.9 (10.0-19.5)		0.005	0.0030
**Neutrophils D2**	-	8.5 (6.0-12.5)	12.6 (8.5-18.1)	12.5 (8.7-18.1)	12.5 (8.7-17.7)	0.0072	0.0032	0.0016
**Lymphocytes D2**	–	0.9 (0.5-1.3)	0.7 (0.5-1.1)	0.8 (0.5-1.2)	0.8 (0.5-1.2)			
**Basophils D2**	–	0.01 (0-0.20)	0.01 (0.00-0.01)	0.01 (0.00-0.10)	0.01 (0.00-0.20)			
**Platelets D2**	–	143 (119-209)	185 (106-247)	159 (103-245)	172 (105-248)			
**CRP D5**	–	138 (89-175)	110 (56-175)	94 (497-163)	105 (53-170)			
**WBC D5**	–	9.8 (7.2-12.4)	13.1 (11-18.1)	11.8 (9.3-16.4)	12.7 (9.6 – 17.2)			
**Neutrophils D5**	-	7.6 (5.7-10.8)	10.7 (8.1-15.1)	9.8 (6.8-14.4)	10.2 (6.9-14.9)	0.0275		0.0475
**Lymphocytes D5**	-	0.8 (0.7-1.2)	1.2 (0.9-1.7)	1.1 (0.7-1.4)	1.1 (0.7 -1.5)	0.0147		0.0383
**Basophils D5**	–	0.01 (0.00-0.01)	0.01 (0.00-0.1)	0.01 (0.00-0.3)	0.01 (0.00-0.10)			
**Platelets D5**	–	173 (157-209)	192 (131-278)	167 (99-253)	178 (114-262)			
**CRP Discharge**	–	80 (55-179)	54 (25-128)	48 (28-124)	51 (26-127)			
**WBC Discharge**	–	10.7 (8.6-12.5)	10.4 (8.1-16.4)	10.1 (8.1-13.0)	10.1 (8.0-14.0)			
**Neutrophils Discharge**	–	8.6 (6.3-10.4)	8.3 (6.5 -12.2)	7.4 (5.8-8.8)	7.6 (5.9-10.6)			
**Lymphocytes Discharge**	–	1.3 (0.8-1.8)	1.4 (1.1-1.7)	1.2 (0.9-2.1)	1.3 (0.9-2.1)			
**Basophils Discharge**	–	0.01 (0.00-0.02)	0.01 (0.00-0.07)	0.02 (0.00-0.10)	0.01 (0.00-0.10)			
**Platelets Discharge**	–	212 (149-284)	269 (166-340)	282 (144-417)	275 (162-355)			

Cell counts for total white blood cells (WBC), neutrophils, lymphocytes, basophils, free platelets and for c-reactive protein (CRP) at days 1, 2, 5 and discharge, plus significance levels between SIRS and individual and combined sepsis groups. CNTRL, Healthy donor controls; SIRS, Out-of-hospital cardiac arrest; SIRS, systemic inflammatory response syndrome; ABDM, abdominal sepsis; PLMN, pulmonary sepsis; ABDM+PLMN, combined sepsis; APACHE II, Acute Physiological and Chronic Health Evaluation score II; SOFA, Sequential Organ Failure Assessment; CRP, C-reactive protein; WBC, White Blood Cell count.

### 3.2 Clinical and hematological parameter statistical analyses

**Figure 2:**
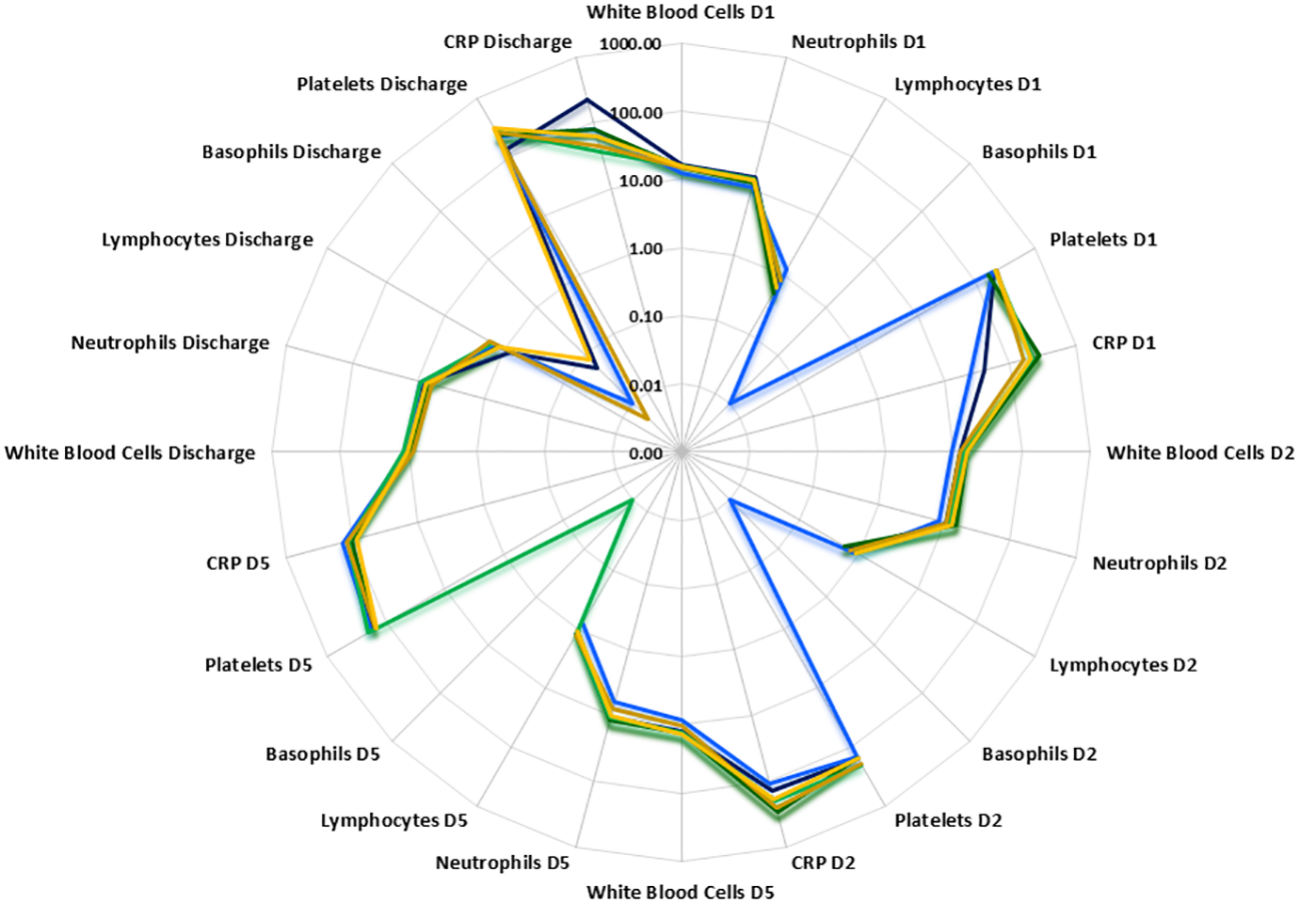
Radar plot of white blood cell, neutrophil, lymphocyte, basophil, free platelet and CRP counts at Days1, 2, 5 and discharge. PLMN DNS 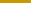, PLMN S 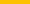, SIRS DNS 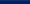, SIRS S 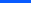, ABDM DNS 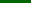, ABDM S 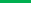.

Temporal differences were observed between disease groups i.e. SIRS and ABDM or PLMN sepsis (**Table 1**) and also when stratified further for patients who survived (S), or did not survive (DNS)) for several clinical parameters (**Figure 2** and **Supplementary Information 1**, **Supplementary Table 1.11** and **Supplementary Table 1.12**). APACHE II scores were calculated on Day1 only and did not show significant variation between the groups. SOFA scores were elevated across all SIRS and sepsis groups on Day 1 indicating multiorgan failure but did not vary significantly between them. SOFA scores and CRP values fell over the trajectory time course of the study in all groups but remained well above normal levels (CRP; > 1.0-2.0 μg/ml (35, 36)), even at the discharge timepoints. White blood cell (WBC) and neutrophil counts were similar across the SIRS and sepsis disease groups at Day1, but significantly higher in the sepsis groups than the SIRS group at Day2. Lymphocyte counts were significantly lower in both the sepsis groups compared with the SIRS group at Day1 only and with the ABDM sepsis group only at Day5. Basophil counts were significantly different between the SIRS and ABDM groups at Day1 only, although generally the counts were low across all groups and timepoints. Free platelet counts showed no significant differences between any disease groups at any timepoint. CRP concentrations were in the pathological range in all groups, but significantly higher in the sepsis groups compared with the SIRS group on Day1. CRP appeared to be a good classifier for Sepsis (compared with SIRS) at this and the Day2 time points. However, at the Day5 and discharge time points, CRP levels were higher in SIRS compared with Sepsis patients and highest at the Discharge timepoint, although these were not statistically significant. When patient groups were stratified for survival i.e. S vs DNS (**Figure 2**) small differences were seen for the SIRS and sepsis S and DNS groups, some of which approached significance/were significant (highlighted in bold); SIRS Day1 [lymphocytes (p = 0.0931), CRP (p = 0.1286)], Day2 (white blood cells (p = 0.0885), neutrophils (p = 0.1206) and basophils (p = 0.033) and Day5 (white blood cells (p = 0.0599), neutrophils (p = 0.0879) and lymphocytes (p = 0.1803). There was an observed 2.76-fold difference in CRP in the SIRS DNS group at the discharge time-point, but this was not statistically significant (due to low group replicates). Differences were seen for the ABDM sepsis group at Day1 [lymphocytes (p = 0.034), basophils (p = 0.1373) and platelets (p = 0.1297)], Day2 [lymphocytes (p = 0.0113), basophils (p = 0.1492)] and discharge timepoints [lymphocytes (p = 0.0323). Small differences were seen for the PLMN sepsis group at the Day5 [CRP (p = 0.0926)] and Discharge timepoints [basophils (p = 0.1516)] only. This reflected slight difference in basophil counts in the PLMN survivors’ group at this latter timepoint.

### 3.3 Microarray data analysis

#### 3.3.1 ANOVA analysis

Analysis of variance (ANOVA) was performed to identify statistically significant, differentially regulated features across disease states with respect to baseline controls on filtered data, applying the Benjamini Hochberg (BH-FDR) multiple testing correction and selecting a cut-off of p ≤ 0.05) across the CNTRL, SIRS, ABDM and PLMN sepsis groups (all time points data included). A large number of statistically-significant biomarkers were identified, 46227 entities remaining after ANOVA, representing 91.13% of all filtered features on the array [data ranked from lowest to highest p-value (**Supplementary Information 2**, **Supplementary Table 2.1**)]. Top and bottom ranked 100 hits for each disease group are given in **Supplementary Information 2**, **Supplementary Table 2.2.** Further fold-change analyses were conducted across all entities and days using default settings (FC > 2.0 (**Supplementary Information 2**, **Supplementary Table 2.3**) on all identified features remaining from ANOVA.

Differential expression of many entities was observed between groups and temporally across timepoints. Top ranked hits included FAM20A, PPARG, ADM and ARG1, many of which are commonly expressed in both SIRS and sepsis disease groups relative to controls and are non-specific. Although there are many common entities shared between the SIRS and sepsis groups, there are also other clear relative expression differences. Gene entities exhibiting stronger expression in the SIRS-ranked dataset included CFC1, CT62, lnc-DAAM2-1 and lnc-LTBP3-2 and in the sepsis-ranked dataset included TDRD9, DAAM2, OLFM4 and OLAH. Previously identified hub markers TDRD9, CD177 and SLC16A3 (25) were represented in the top twenty-five ranked hits and KLRK1, GPR84, PCOLCE2 in the top three hundred. The remaining hub markers MYL9 and FGF13 ranked somewhat lower in the top four thousand and may be components of other, more distinct, disease-specific responses. Other previously identified genes, which also featured highly significantly in this dataset, included ARG1, METTL7B, and RETN. These may represent components of a non-specific severe inflammatory response from commonly represented cell types, probably a generalized ‘emergency-response’ module. Data were stratified according to disease group, timepoint and survival [(S)/(DNS)] and cluster analysis conducted for these select biomarkers (**Supplementary Information 3**, **Supplementary Figure 3.1**). Many of the inflammatory biomarkers highlighted above were found to be temporally expressed over the time course of the study.

#### 3.3.2 Principal component analysis (PCA) and identification of biomarkers of severe inflammation for primary admission assessment

**Figure 3:**
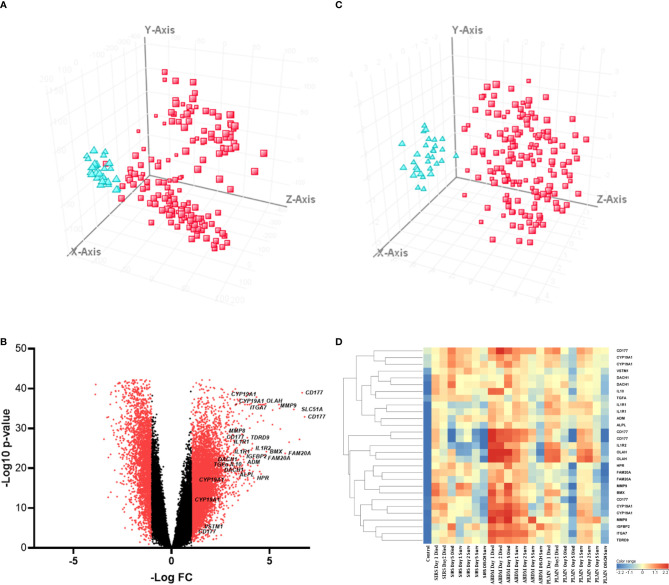
**(A)** PCA analysis of CNTRL 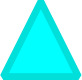 versus combined SIRS&Sepsis biomarker groups 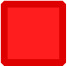 (each symbol depicting an individual within each group) **(B)** volcano plot of log_10_ p-value vs log fold-change of all gene entities, using a 2-fold change cutoff and with select I°I genes highlighted **(C)** PCA analysis of CNTRL 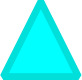 versus combined SIRS&Sepsis biomarker groups 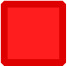 (each symbol depicting an individual within each group) using select I°I genes only (from **Table 2**) **(D)** heat map of select I°I biomarkers from **Table 2** across all control, SIRS, ABDM and PLMN sepsis groups stratified by day and prognosis (died/survived).

To identify significantly differentially expressed, entities at an early ICU admission timepoint, PCA was performed comparing CNTRL vs SIRS and Sepsis combined [(Combined) **Figure 3A** and **Supplementary Information 2**, **Supplementary Table 2.4**]. Fold-change expression values (>2.0 and adjusted p-value p < 0.001) were then conducted across combined timepoints (**Supplementary Information 2**, **Supplementary Table 2.5**) and the Day1 timepoint only (**Supplementary Information 2**, **Supplementary Table 2.6**) to identify those with the most likely discriminatory power for use in a diagnostic and primary contact setting. CD177, ARG1, FAM20A, PCOLCE2, SLC51A, MMP9, were identified as most significantly upregulated in both SIRS and Sepsis on Day1, compared with healthy controls, with CD177 and ARG1 consistently higher for both SIRS and Sepsis at this and across all timepoints to discharge. DAAM2 and OLAH were significantly upregulated in both SIRS (FC>8) and sepsis (FC>20) compared to controls, but approximately 3-fold higher in sepsis, than SIRS. This suggests that although the majority of these biomarkers are differentially regulated in both conditions, there are subtle differences. Biomarkers were selected for further progression using a combination of factors including P-cov, p-value and positive fold-change compared with healthy controls (**Figure 3B**) and empirical quality assessment (**Table 2**) These were named Indicators of Inflammation (I°I) which in combination show clear, improved resolution of healthy controls and combined SIRS/sepsis disease groups (**Figure 3C** and depicted in heatmap format in **Figure 3D**).

**Table 2:** IoI single biomarker gene analyses, ranked by AUC value derived ROC analysis at the Day1 timepoint with p-value <0.0001 and cut-offs selected to obtain optimal sensitivity and specificity and positive and negative predictive values (PPV/NPVs).

Gene	Regulation	Probe	FC (Day1)	AUC	Cutoff >	Sensitivity %	Specificity %	LR +	LR -	PPV	NPV
*ADM*	up	A_23_P127948	12.95	0.9988	-1.928	98.80 (96.28-99.79)	96.67 (85.14-99.83)	29.67	0.01	99.40	93.55
*FAM20A*	up	A_32_P108254	55.35	0.9982	-2.339	97.60 (94.60-99.18)	96.67 (85.14-99.83)	29.31	0.02	98.76	77.78
*ITGA7*	up	A_23_P128084	15.67	0.9968	-2.314	95.81 (92.27-98.02)	96.67 (85.14-99.83)	28.77	0.04	99.38	80.56
*FAM20A*	up	A_24_P352952	26.69	0.9950	-1.842	94.01 (90.05-96.72)	96.67 (85.14-99.83)	28.23	0.06	100.00	78.95
*TDRD9*	up	A_32_P208350	14.53	0.9914	-1.756	93.41 (89.33-96.26)	96.67 (85.14-99.83)	28.05	0.07	99.36	75.00
*IL10*	up	A_23_P126735	7.06	0.9912	-0.674	89.22 (84.44-92.92)	96.67 (85.14-99.83)	26.79	0.11	99.33	61.70
*MMP9*	up	A_23_P40174	45.49	0.9906	-3.073	95.81 (92.27-98.02)	96.67 (85.14-99.83)	28.77	0.04	99.38	80.56
*CD177*	up	A_23_P259863	110.31	0.9876	-1.891	88.02 (83.07-91.92)	96.67 (85.14-99.83)	26.43	0.12	99.31	53.85
*BMX*	up	A_23_P253602	28.57	0.9816	-2.417	94.61 (90.78-97.16)	96.67 (85.14-99.83)	28.41	0.06	99.37	76.32
*CD177*	up	A_21_P0011751	100.99	0.9804	-1.839	85.63 (80.39-89.89)	96.67 (85.14-99.83)	25.71	0.15	100.00	56.60
*HPR*	up	A_23_P206760	18.26	0.9766	-2.407	93.41 (89.33-96.26)	96.67 (85.14-99.83)	28.05	0.07	99.36	72.50
*DACH1*	up	A_33_P3316786	4.58	0.9756	-1.209	94.61 (90.78-97.16)	96.67 (85.14-99.83)	28.41	0.06	99.37	78.95
*CYP19A1*	up	A_23_P37410	9.68	0.9729	-1.069	86.83 (81.73-90.91)	96.67 (85.14-99.83)	26.08	0.14	99.32	56.86
*DACH1*	up	A_23_P32577	5.73	0.9729	-1.649	95.81 (92.27-98.02)	90.00 (76.14-97.22)	9.58	0.05	98.16	79.41
*IGFBP2*	up	A_23_P119943	12.73	0.9695	-1.533	85.63 (80.39-89.89)	96.67 (85.14-99.83)	25.71	0.15	99.30	54.72
*ALPL*	up	A_24_P353619	9.86	0.9685	-1.383	86.23 (81.06-90.40)	96.67 (85.14-99.83)	25.89	0.14	99.31	55.77
*OLAH*	up	A_23_P161458	26.15	0.9633	-2.885	90.42 (85.81-93.90)	96.67 (85.14-99.83)	27.15	0.10	99.34	64.44
*CYP19A1*	up	A_33_P3351371	7.34	0.9627	-0.888	85.03 (79.72-89.37)	96.67 (85.14-99.83)	25.53	0.15	100.00	54.55
*ILR1*	up	A_33_P3396389	8.06	0.9495	-1.821	90.42 (85.81-93.90)	90.00 (76.14-97.22)	9.04	0.11	100.00	54.55
*OLAH*	up	A_33_P3317109	9.98	0.9441	-1.493	85.63 (85.81-93.90)	96.67 (85.14-99.83)	25.71	0.15	99.31	56.60
*ILR1*	up	A_24_P200023	8.25	0.9421	-1.525	86.83 (85.81-93.90)	96.67 (85.14-99.83)	26.08	0.14	99.32	56.86
*MMP8*	up	A_23_P24493	6.77	0.9329	-1.608	85.63 (85.81-93.90)	86.67 (72.04-95.31)	6.42	0.17	97.96	52.00
*TGFα*	up	A_23_P377291	3.93	0.9327	-0.945	85.03 (79.72-89.37)	83.33 (68.00-93.19)	5.10	0.18	42.65	42.65
*IL1R2*	up	A_24_P63019	17.29	0.9323	-1.928	85.63 (85.81-93.90)	93.33 (80.47-98.80)	12.84	0.15	88.62	53.85
*CD177*	up	A_33_P3232080	6.23	0.9104	-1.303	85.03 (79.72-89.37)	90.00 (76.14-97.22)	8.50	0.17	98.62	46.15
*CYP19A1*	up	A_24_P920646	2.36	0.8467	-0.538	85.03 (79.72-89.37)	46.67 (30.85-63.01)	1.59	0.32	89.81	35.00
*CYP19A1*	up	A_32_P86289	2.03	0.8178	-0.552	85.63 (85.81-93.90)	43.33 (27.87-59.84)	1.51	0.33	90.63	35.14
*VSTM1*	up	A_33_P3514487	2.83	0.7721	-1.483	85.63 (85.81-93.90)	40.00 (24.95-56.61)	1.43	0.36	88.82	33.33
*CD177*	up	A_33_P3232086	2.24	0.7621	-0.544	85.63 (85.81-93.90)	43.33 (27.87-59.84)	1.51	0.33	89.94	34.21

#### 3.3.3 T-Test analysis; delineation of biomarkers of clinical outcome/prognosis

Patients who died or survived (sepsis and SIRS combined) were compared with T-tests. This confirmed prominence of ARG1 and another immunosuppressive cytokine IL10 with a poor prognosis/outcome (**Supplementary Information 2**, **Supplementary Table 2.7** – upregulated in patients who died), among others. Biomarkers associated with a good prognosis were also identified (**Supplementary Information 2**, **Supplementary Table 2.8** – upregulated in patients who survived) e.g., CCR9, CD27, LTK and LTB (TNFβ), among others. This suggests correlation of certain biomarkers associated with a more immunosuppressive phenotype (i.e., IL10) with poor outcomes and other more pro-inflammatory immune response biomarkers (i.e., TNFβ) with good outcomes. Other biomarkers also correlate with outcome/prognosis e.g., CD177, FGF13, GRB10 and PPARG (**Supplementary Information 3**, **Figure S3.2**) in both SIRS and sepsis.

#### 3.3.4 Primary identification of disease-specific response genes

**Figure 4:**
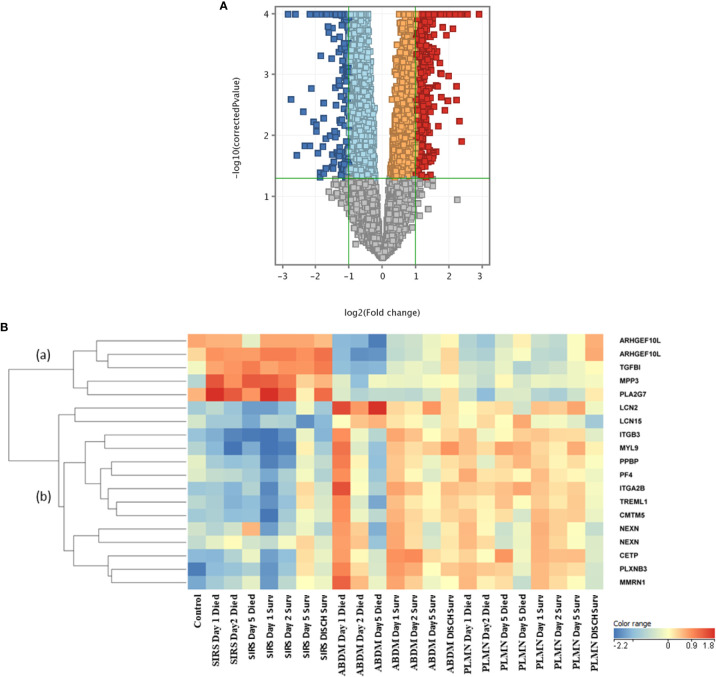
**(A)** Volcano plot of T-Test results from analysis of SIRS versus Sepsis biomarker groups **(B)** heat map of select S°S biomarkers from **Table 3** across all control, SIRS, ABDM and PLMN sepsis groups stratified by day and prognosis (died/survived).

To identify genes which may discriminate between SIRS and sepsis, normalized data stratified on disease group were compared using T-tests (T-test volcano plot depicted in **Figure 4A**). Many entities were found which discriminated between SIRS (**Supplementary Information 2**, **Supplementary Table 2.9** (upregulated in SIRS)) and sepsis (**Supplementary Information 2**, **Supplementary Table 2.10** (upregulated in Sepsis)). SIRS and sepsis-specific biomarkers were found to distinguish between SIRS and both ABDM and PLMN sepsis sub-types. Biomarkers were also selected for further progression using a combination of factors including Pcov, p-value, fold change and empirical quality assessment (**Table 3**). These were termed SIRS or Sepsis indicators (S°S), showed varying patterns of expression between SIRS and sepsis groups (**Figure 4B**) and dysregulated, temporal patterns of expression across the time-course of the study.

**Table 3:** SIRS or sepsis (SoS) single biomarker gene analyses, ranked by AUC value derived ROC analysis at the Day1 timepoint with p-value <0.0001 and cut-offs selected to obtain optimal sensitivity and specificity and positive and negative predictive values (PPV/NPVs).

Genes	Regulation Sepsis vs SIRS	Probe	FC (Day1)	AUC	Cutoff >	Sensitivity %	Specificity %	LR +	LR -	PPV	NPV
*CETP*	Up	A_23_P49376	4.95	0.91	-0.64	83.87 (77.43-89.05)	71.79 (57.66-83.31)	2.97	0.22	90.35	57.14
*ITGB3*	Up	A_24_P318656	7.47	0.87	-0.94	85.48 (79.24-90.40)	71.79 (57.66-83.31)	3.03	0.20	90.60	63.04
*NEXN*	Up	A_33_P3341429	4.24	0.86	-0.51	83.87 (77.43-89.05)	76.92 (63.18-87.40)	3.63	0.21	92.04	60.00
*PLXNB3*	Up	A_33_P3413038	5.10	0.85	-0.59	83.06 (76.54-88.36)	81.58 (68.17-91.02)	4.51	0.21	92.45	57.63
*CMTM5*	Up	A_23_P106042	4.93	0.85	-0.81	85.48 (79.24-90.40)	64.10 (46.69-76.83)	2.38	0.23	88.33	58.14
*MMRN1*	Up	A_33_P3212257	4.66	0.85	-0.59	83.06 (76.54-88.36)	82.05 (68.92-91.26)	4.63	0.21	93.64	83.78
*ITGA2B*	Up	A_24_P65373	4.89	0.84	-1.37	85.48 (79.24-90.40)	64.10 (46.69-76.83)	2.38	0.23	88.33	58.14
*PF4*	Up	A_24_P79403	3.59	0.84	-0.66	85.48 (79.24-90.40)	61.54 (47.11-74.59)	2.22	0.24	87.60	57.14
*MYL9*	Up	A_23_P210425	5.55	0.83	-1.53	85.48 (79.24-90.40)	61.54 (47.11-74.59)	2.22	0.24	87.60	59.52
*PPBP*	Up	A_23_P121596	4.36	0.83	-0.94	85.48 (79.24-90.40)	58.97 (44.57-72.31)	2.08	0.25	87.50	55.81
*TREML1*	Up	A_33_P3381777	4.10	0.82	-1.35	85.48 (79.24-90.40)	48.72 (34.72-62.86)	1.67	0.30	84.13	56.76
*LCN2*	Up	A_23_P169437	3.60	0.82	-1.24	85.48 (79.24-90.40)	61.54 (47.11-74.59)	2.22	0.24	88.33	57.14
*NEXN*	Up	A_23_P200001	2.37	0.82	-0.24	82.26 (75.64-87.67)	66.67 (52.31-79.03)	2.47	0.27	88.70	54.17
*LCN15*	Up	A_33_P3263938	2.02	0.68	-2.52	85.48 (79.24-90.40)	20.51 (10.64-33.98)	1.08	0.71	77.37	30.77
*PLA2G7*	Down	A_23_P145096	-7.09	0.90	1.00	85.48 (79.24-90.40)	82.05 (68.92-91.26)	4.76	0.18	64.00	93.81
*MPP3*	Down	A_23_P141345	-3.28	0.89	0.67	82.05 (68.92-91.26)	83.87 (77.43-89.05)	5.09	0.21	61.54	93.69
*ARHGEF10L*	Down	A_33_P3799936	-3.86	0.88	0.64	85.48 (79.24-90.40)	66.67 (52.31-79.03)	2.56	0.22	59.09	86.55
*ADGRA2/GPR124*	Down	A_23_P43276	-3.34	0.86	0.93	85.48 (79.24-90.40)	64.10 (46.69-76.83)	2.38	0.23	58.14	88.33
*APCDD1*	Down	A_23_P337262	-3.66	0.84	0.33	87.18 (74.91-94.81)	66.13 (58.50-73.17)	2.57	0.19	44.74	94.25
*ARHGEF10L*	Down	A_33_P3215575	-2.81	0.81	-0.20	87.18 (74.91-94.81)	64.06 (56.50-71.13)	2.43	0.20	51.25	94.25
*MYCL*	Down	A_33_P3306068	-2.95	0.81	0.51	85.94 (79.86-90.71)	66.67 (52.31-79.03)	2.58	0.21	36.95	93.33

Expression of the SIRS-associated biomarkers appeared broadly unchanging in the SIRS group and did not correlate with time or prognosis (sub-cluster (a)). However, these markers correlated well with a prognosis/recovery in the sepsis groups, particularly ARHGEF10L and PLA2G7. Expression of sepsis-associated biomarkers in subclusters (b) and (c) were relatively high across the sepsis group timepoints, with some variation, but again did not correlate with prognosis. These sepsis-associated biomarker gene lists are particularly enriched for platelet and megakaryocyte-associated entities e.g., ITGA2B, ITGB3, GP6, MPIG6B, MYL9, PF4, PPBP and SELP etc. Increased expression of some of these was observed in the SIRS group at Day5 e.g., ITGA2B, which may indicate development of sepsis-like characteristics, perhaps indicative of emerging infection. Expression was reduced at the discharge time-point in the SIRS survivor group.

#### 3.3.5 Validation of disease-specific response genes using ANN analysis

A stepwise artificial neural network modeling analysis (ANN) was used to predict the best discriminatory genes between the SIRS and sepsis groups using select candidate biomarker genes from the ANOVA analysis. ANN confirmed importance of the SIRS/sepsis discriminatory biomarkers, including ARHGEF10L, PLA2G7, PLXNB3, MPP3 and CETP for discriminating SIRS from ABDM (**Supplementary Information 2**-**Supplementary Table 2.11**) and PLMN sepsis (**Supplementary Information 2**-**Supplementary Table 2.12**). Similar analyses using Day1 samples only confirmed ARHGEF10L, PLA2G7, PLXNB3 and CETP but not MPP3 to be discriminatory at this crucial early/first contact timepoint for ABDM (**Supplementary Information 2**-**Table S2.13**) and PLMN sepsis (**Supplementary Information 2**-**Supplementary Table 2.14**). CD38 and NID2 were the primary markers found to discriminate PLMN from ABDM (**Supplementary Information 2**-**Supplementary Table 2.15**). Other secondary discriminatory biomarkers included those previously noted e.g., ITGA2B and also GPR124, SPOCD1, MMRN1, SAMD14, GPR124, and SELP, where expression is somewhat higher in the ABDM with regard to the PLMN group, particularly in the non-survivor group, at Day1 (**Supplementary Information 4**, **Supplementary Figure 4.1**).

#### 3.3.6 Selection of biomarker signatures using random forest modeling

**Figure 5:**
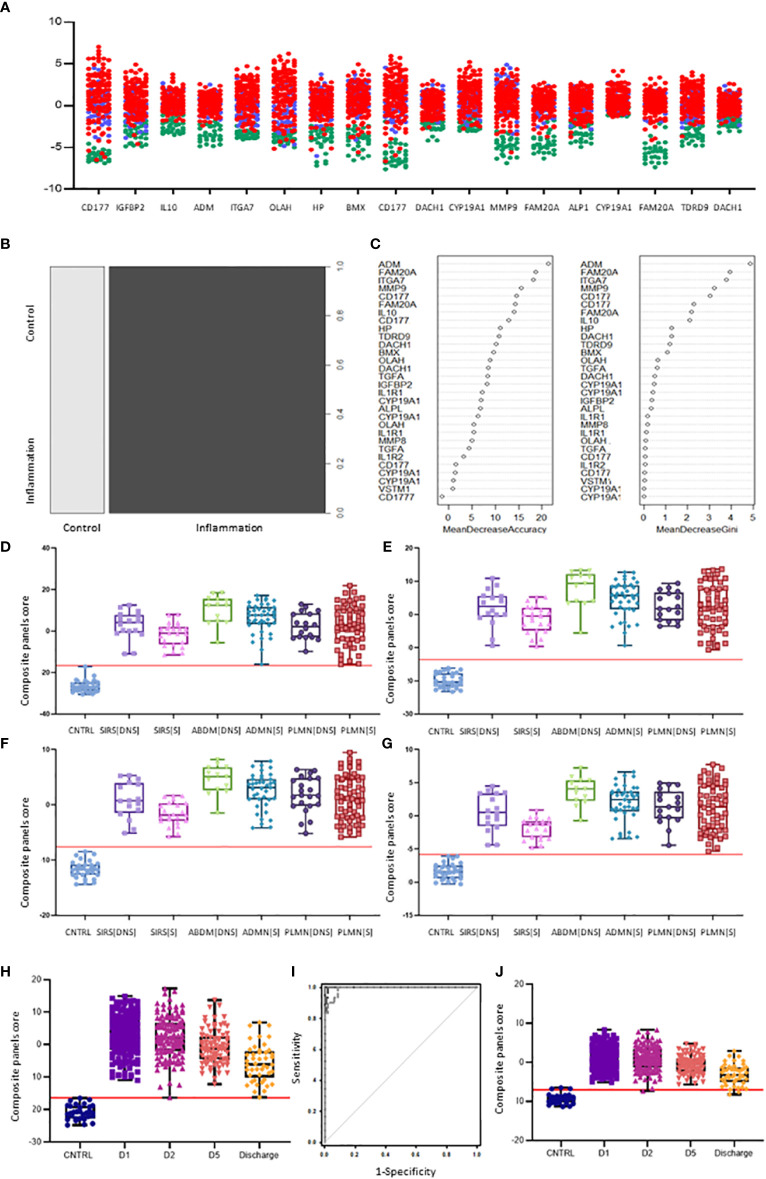
Selection of I°I signatures **(A)** Dot plot depiction of individual inflammatory biomarker candidates, including data from multiple biomarker probes where present. CNTRLs 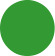, SIRS 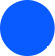, Sepsis 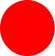 **(B)** Random forest classification of validation data into controls and inflammation groups with ‘mtry’ of 31, ‘ntree’ of 2001 **(C)** Visualization of random forest models features of importance ranked by mean decrease accuracy and mean decrease Gini score **(D)** I°I candidate panel: ADM+CD177+FAM20A+ITGA7+MMP9+OLAH **(E)** I°I candidate panel: ADM+FAM20A+ OLAH+ITGA7+MPP9 **(F)** I°I candidate panel: ADM+OLAH+ FAM20A **(G)** I°I candidate panel: OLAH+FAM20A **(H)** I°I candidate panel: ADM+FAM20A+ OLAH+ITGA7+MMP9 across all time-points **(I)** ROC curves of ADM+FAM20A+OLAH ITGA7+MMP9 and OLAH+FAM20A comparing CNTRL vs SIRS/sepsis across day 1, day 2, day 5 and discharge time points **(J)** I°I candidate panel: OLAH+FAM20A across all timepoints.

Nineteen I°I biomarkers upregulated in both SIRS and Sepsis and twenty S°S biomarkers differentially regulated between SIRS and Sepsis were selected for further study. Performance of individual I°I and S°S biomarkers at the Day1 admission timepoint were assessed by ROC analysis (**Tables 2**, **3**). The I°I biomarkers showed outstanding performance, with many achieving excellent AUC values: >0.99 90% CI 0.9104-0.9988 **Table 2** and **Figure 5A**. Using Day1 admission timepoints only, Random Forest modeling generated an out of bag (OOB) estimate of error rate of 0% for the I°I biomarkers, (**Figure 5B**), predicting 100% accuracy in classification of samples and ranking ADM, FAM20A, ITGA7, MMP9 and CD177 as most important, by both Mean Decrease Accuracy and Gini scores (**Figure 5C**). To identify the most significant inflammatory biomarkers upregulated throughout the duration of ICU stay, Random Forest modeling was performed on all timepoints with an OOB estimate of error rate of 0.88% predicted on the training set. ITGA7, ADM, FAM20A, TDRD9, MMP9, CD177, IL10 were all consistently ranked of highest importance. Classification of data split into three separate groups: Controls, SIRS and Sepsis achieved an OOB estimate of error rate of 17.99% across all days, revealing three biomarkers of most importance by feature selection i.e., FAM20A, OLAH and DAAM2.

**Figure 6:**
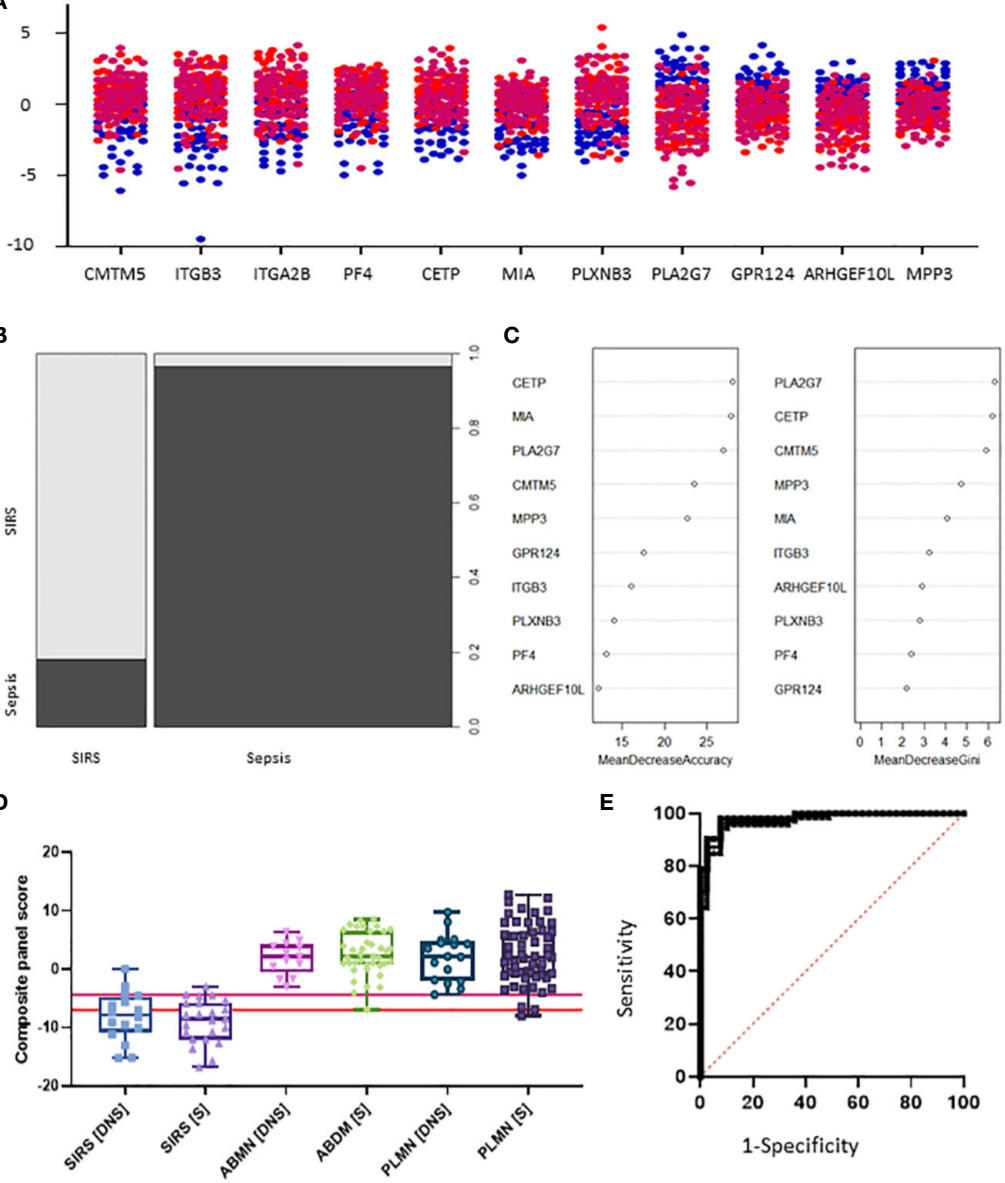
Selection of S°S signatures **(A)** Dot pot depiction of individual SIRS/sepsis discriminatory biomarker candidates, with multiple versions of probes for some biomarkers. SIRS 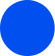, ABDM 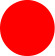, PLMN 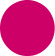. **(B)** Random forest classification of validation data into SIRS and Sepsis ‘mtry’ of 11, ‘ntree’ of 2001 **(C)** Visualization of random forest models features of importance ranked by mean decrease accuracy and mean decrease Gini score **(D)** S°S candidate panel: CETP+CMTM5+MIA-MPP3-PLA2G7 **(E)** ROC curves of CETP+CMTM5+MIA-MPP3-PLA2G7 for SIRS vs Sepsis, SIRS vs Abdominal sepsis and SIRS vs Pulmonary sepsis comparisons.

Candidate S°S biomarkers also performed well with good AUC values (>0.84 90% CI 0.6756-0.9069) **Table 3** and **Figure 6A**). Reflecting the likely clinical diagnostic requirement for differentiation of Sepsis from SIRS, biomarker signatures were sought that could identify both ABDM and PLMN sepsis and which could discriminate those from the SIRS group with a high degree of accuracy at Day1 of ICU admission. Random Forest modeling was again performed using Day1 timepoint data only, initially using a large selection of entities and repeatedly run with the least important entities removed iteratively from each model run. A final model with a filtered selection of 10 entities (PLA2G7, ARHGEF10L, CMTM5, ITGB3, CETP, MIA, PLXNB3, MPP3, GPR124, PF4) achieved an OOB error rate of 7.38% (**Figures 6B, C**) and ranked CETP, MIA, PLA2G7, CMTM5 and MPP3 of greatest importance by Mean decrease Accuracy and Gini score. Biomarkers of most importance varied with each repeated model and between SIRS and ABDM or PLMN sepsis, suggesting subtle differences between groups.

### 3.4 Selection of I°I biomarker panels using simple algorithms and performance assessment via ROC analysis

**Figure 7:**
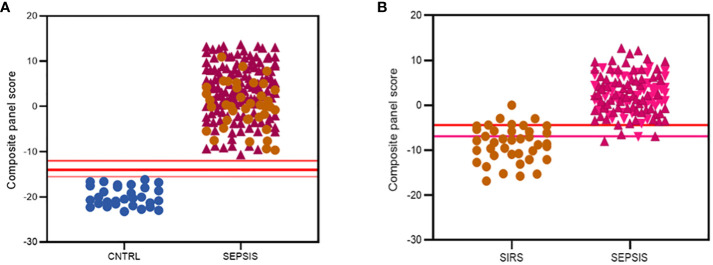
Summary of best performing signatures and cut-off values to maximize discriminatory performance of **(A)** the I°I signature; CNTRL 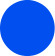, SIRS 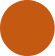, SEPSIS 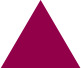 **(B)** the S°S Signature; SIRS 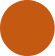, PLMN SEPSIS 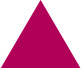, ABDM SEPSIS 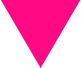.

Various combinations of I°I biomarkers were assessed manually as simple additive algorithms, calculated using composite panel scores to determine which combination best discriminated disease and control groups. Using ROC analysis, many I°I biomarker combinations were able to achieve an AUC value of 1.0 at Day1 of admission e.g. (a) ADM+CD177+FAM20A+ITGA7+MMP9+OLAH (b) ADM+FAM20A+OLAH+ITGA7+MPP9 (c) ADM+ OLAH+FAM20A (d) OLAH+FAM20A. These results were also obtained using data stratified into S or DNS SIRS and Sepsis groups (**Figures 5D-G**). Further analysis and alternate combinations are available in **Supplementary Information 5**, **Supplementary Table 5.1**). Significant differences between S and DNS were observed between SIRS and ABDM but not PLMN sepsis for small signatures: FAM20A+OLAH, ADM+FAM20A+OLAH (p <0.05). No significant differences were seen when using the large I°I panel ADM+CD177+FAM20A+ITGA7+MPP9+OLAH. Although, all signatures depicted achieved an AUC of 1.0, variation in separation between controls and SIRS/sepsis groups and cut-offs is visible within the panels with the largest combination of biomarkers i.e. (D) ADM+CD177+FAM20A+ITGA7+MPP9+OLAH showing smallest differences between control and disease groups. With CD177 removed (E), the panel appears to show best performance with greatest separation between groups, although the smaller panels of FAM20A+OLAH perform almost as well. Both combinations distinguished inflammation from controls with an AUC >0.99 across all days, including discharge (**Figures 5H-J**) and showed good separation between controls and all Sepsis and SIRS groups. A cut-off value of -14.0 was selected for panel ADM+FAM20A+ITGA7+MPP9+OLAH for discrimination of SIRS and sepsis groups from controls (**Figure 5H**), which provides a positive predictive value (PPV) and negative predictive value (NPV) each of 100%. This cut-off could be placed anywhere between -12.0 and -15.5 for this data and show 100% accuracy in classification of the disease from control groups (**Figure 7A**).

### 3.5 Selection of S°S biomarker panels using simple algorithms and performance assessment via ROC analysis

Simple additive algorithms were also composed manually for the S°S signature biomarker combinations (added or subtracted dependent on upregulation or downregulation in sepsis), to calculate a composite panel score from which diagnostic accuracy of the combined biomarker signatures could be assessed using ROC analysis. Best performing signatures were selected based on their PPVs and NPVs, with the aim of selecting combinations and corresponding cut-off values to detect sepsis with high performance i.e. PPV of >95% or to rule out Sepsis with a NPV >98% (35), based on those described for disease with similar prevalence (36). Many combinations of biomarkers showed excellent discrimination of Sepsis from SIRS (**Supplementary Information 5**, **Supplementary Table 5.2**). A 5-biomarker signature of the top-ranking Random Forest predicted biomarkers: CETP+CMTM5+MIA-MPP3-PLA2G7 showed the best discriminatory performance for SIRS and Sepsis combined with an AUC of 0.9758 (90% CI: 0.9582-0.9933) and individually for ABDM and PLMN with AUCs of 0.9842 (90% CI: 0.9864-1.00) and 0.9698 (90% CI: 0.9468-0.9928), as shown in **Table 4** and **Figures 6D, E**. Two cut-offs were selected to optimize diagnosis with a ‘ruling-out’ sepsis cut-off of -4.3770, which provided a PPV of 96.95% and a NPV of 89.74%, equivalent to three false positive patients and five false negatives, out of 164 total Day1 samples (**Figure 7B**). A second cut-off was selected at -6.980 which generated a PPV of 90.27% and NPV of 96.15% which predict patients at high risk of having sepsis.

**Table 4:** ROC analysis results SoS signature: CETP+CMTM5+MIA-MPP3-PLA2G7 comparing SIRS and Sepsis, then SIRS and abdominal and pulmonary sepsis and corresponding cut-off values selected to exemplify 95% PPV and 98% NPV.

Comparison	SIRS vs Sepsis	SIRS vs Abdominal sepsis	SIRS vs Pulmonary sepsis
Panel	*CMTM5+CETP-PLA2G7-MIA-MPP3*	*CMTM5+CETP-PLA2G7-MIA-MPP3*	*CMTM5+CETP-PLA2G7-MIA-MPP3*
AUC	0.9758	0.9842	0.9698
Standard Error	0.01067	0.009643	0.01398
90% CI	0.9582 - 0.9933	0.9684 - 1.000	0.9468 - 0.9928
p-value	< 0.0001	< 0.0001	< 0.0001
95% PPV Cut-off >	-4.3770	-2.5610	-4.1520
Sensitivity (CI%)	96.8000	90.3800	94.5200
Specificity (CI%)	89.7400	97.4400	92.3100
LR +	9.4347	35.3047	12.2913
LR -	0.0357	0.0987	0.0594
PPV	97.60	97.92	96.00
NPV	89.74	88.37	92.11
98% NPV Cut-off >	-6.9830	-4.1670	-6.9570
Sensitivity (CI%)	99.2000	98.0800	98.6300
Specificity (CI%)	64.1000	92.3100	64.1000
LR +	2.7632	12.7542	2.7474
LR -	0.0125	0.0208	0.0214
PPV	89.85	94.44	84.88
NPV	96.15	97.30	96.15

**Figure 8:**
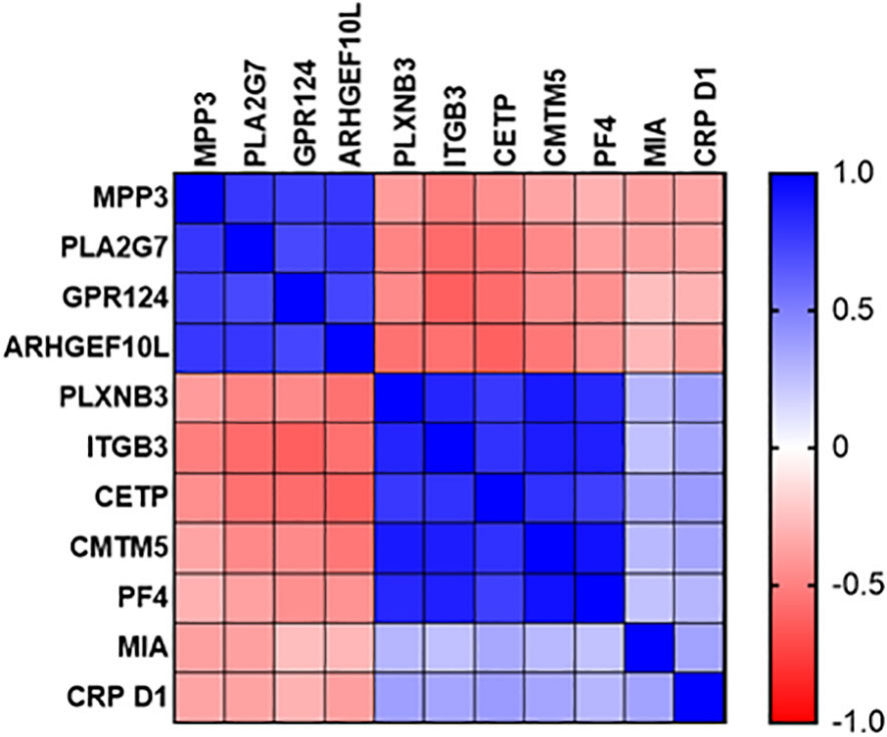
Correlation plot of diagnostic performance of SIRS and sepsis-specific biomarkers to each other and to CRP.

Expression of sepsis-specific biomarkers PLXNB3, ITGB3, CETP, CMTM5 and PF4 correlated positively with each other at the Day1 time point (**Figure 8**) and to a slightly lesser degree with MIA and CRP. SIRS-specific biomarkers MPP3, PLA2G7, GPR124 and ARHGEF10L correlated positively with each other and negatively with the sepsis-specific biomarkers and CRP.

### 3.6 Evaluation of I°I signatures on independent previously published datasets

The performance of I°I candidate signatures: ADM+CD177+FAM20A+ITGA7+MPP9+OLAH, ADM+FAM20A+OLAH and FAM20A+OLAH were compared on a wider cohort of samples, five independent, previously published, adult datasets were selected (four Sepsis datasets: GSE154918, GSE131761, GSE28750, GSE65682 and a COVID-19 study which contained a bacterial infection group: GSE16173. Not all candidate signatures could be evaluated on all identified datasets due to inconsistencies e.g., missing entities, discordance with patient group, small sample size or lack of data.

ROC curve analyses were performed, comparing control and disease groups in the available datasets. Good performance was shown for most sepsis vs control comparisons and for identifying a bacterial infection group from healthy control, COVID-19, other coronavirus (CoV) and Influenza viral infection groups (**Supplementary Information 5**-**Supplementary Table 5.3**). Accuracy was reduced for all signatures between 0.80-0.8184 when comparing ABDM sepsis to a gastro-intestinal control group using the GSE65682 dataset. Both I°I signatures performed poorly in recognizing viral infections from healthy controls (GSE161731), suggesting these are not useful for recognizing severe inflammation in viral diseases.

### 3.7 Evaluation of S°S signatures on independent previously published datasets

**Figure 9:**
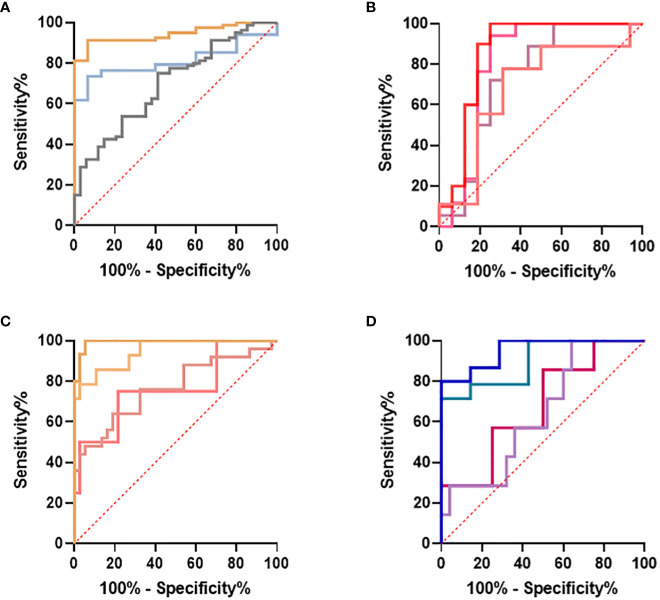
Evaluation of I^O^I Signature (CMTM5+ITGB3-PLA2G7-GPR124-ARHGEF10L) performance on published datasets **(A)** GSE131761 comparing healthy controls and septic shock 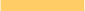, healthy controls and non-septic shock 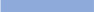, non septic shock and septic shock 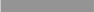 **(B)** GSE9960 comparing healthy controls and sepsis (mixed infection) 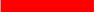, healthy controls and sepsis (gram positive) 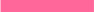, healthy controls and sepsis (gram negative) 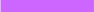 healthy controls and sepsis 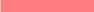 **(C)** GSE154918 comparing healthy controls and sepsis 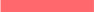, healthy controls and follow up of sepsis 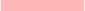, healthy controls and septic shock 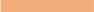 healthy controls and follow up of septic shock 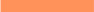 **(D)** GSE154918 comparing uncomplicated infection and sepsis 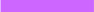, uncomplicated infection and follow up of sepsis 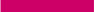, uncomplicated infection and septic shock 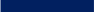, uncomplicated infection and follow up of septic shock 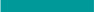.

Similarly, the S°S Signature CETP+CMTM5+MIA-MPP3-PLA2G7 was evaluated using five Sepsis datasets: GSE154918, GSE131761, GSE9960, GSE28750 and GSE65682 alongside other biomarker combinations performing well for ABDM sepsis (CMTM5+ITGB3-ARHGEF10L-GPR124-PLA2G7); PLMN sepsis (CETP+MIA+PLXNB3-MPP3) and two larger panels combining 8 of the best performing biomarkers (CMTM5+ITGB3-PLA2G7-ARHGEF10L-GPR124+CETP+MIA-MPP3) and (CMTM5+ITGB3-PLA2G7-ARHGEF10L+CETP+MIA+PLXNB3-MPP3), given in **Supplementary Information 5**-**Supplementary Table 5.4**. Performance of the S°S signature in discriminating Sepsis from non-sepsis groups across these datasets was highly variable, perhaps impacted by differing study design, patient recruitment, sample collection and technological platform. In GSE28750 our candidate signature of CETP+CMTM5+MIA-MPP3-PLA2G7 performed best in identifying sepsis from post-surgical patients with an AUC of 0.8182 but did not rank highest when analyzed on any other datasets. In GSE154918 significant differences in performance were observed between different biomarker combinations with CMTM5+ITGB3+PLA2G7-GPR124-ARHGEF10L achieving AUC values of 0.9524-0·9928 when comparing Septic Shock to non-sepsis infection and healthy controls respectively. For GSE65682, only candidate signature CMTM5+ITGB3-PLA2G7-GPR124-ARHGEF10L could be evaluated due to missing entities. An AUC of 0.9855 was achieved when comparing ABDM sepsis to healthy controls, reduced to an AUC of 0.7035 when comparing ABDM sepsis to hospital acquired pneumonia. For GSE154918, an AUC of 0.9619 was achieved when comparing septic shock to uncomplicated infection which again reduced to an AUC of 0.7488 on comparison to sepsis only. Of all candidate S°S biomarker combinations trialed on other data sets, ITGB3+CMTM5-PLA2G7-ARHGEG10L-GPR124 showed best performance with AUC values ranging from 0.9928-0.7026 across datasets and group comparisons as summarized in **Figure 9** with highest AUC values obtained when comparing healthy controls to septic shock. When evaluated on GSE131761, this candidate signature achieved an AUC of >0.94 for discriminating septic shock from healthy controls and an AUC of >0.72 for discriminating non-septic shock and septic shock (**Figure 9A**. In GSE9960, CMTM5+ITGB3+PLA2G7-GPR124-ARHGEF10L performed best when comparing healthy controls and sepsis caused by mixed infection or gram-positive infection (**Figure 9B**) with reduced performance for sepsis caused by gram-negative infections. In GSE154918, the candidate S°S signature combination showed good performance in distinguishing sepsis and septic shock from healthy controls (**Figure 9C** with reduced performance observed for uncomplicated infections (**Figure 9D**. It is anticipated that S°S biomarkers could be substituted in and out of S°S signatures to maximize performance and enable effective patient diagnosis according to end user needs. Other biomarker combinations identified could also be suitable for diagnostic progression.

### 3.8 Evaluation of published competitor signatures on the ANEMONES dataset

Five published competitor signatures used for discrimination of Sepsis from SIRS from other groups were evaluated on our dataset: Septicyte Lab (16), FAIM3/PLAC8 ratio (22), sNIP score (23), Bauer gene expression score (37) and Sepsis Metascore (12, 21, 24). (**Supplementary Information 5**, **Supplementary Table 5.5**). Only 2 of 5 of the signatures trialed showed significant discrimination of Sepsis from SIRS within our clinical cohort. Septicyte lab (PLAC8-PLA2G7+LAMP1-CEACAM4) performed best with an AUC value of 0·8377. The Bauer Gene expression score (TLR5, CD59, CLU, FGL2, IL7R, HLA-DPA1, CPVL) achieved an AUC value of 0.7877.

## 4 Discussion

Distinguishing sepsis from other severe inflammatory conditions with significant organ dysfunction is major challenge on the ICUs. Bedside clinicians continue to utilize biomarkers such as CRP and procalcitonin, in addition to more traditional clinical and laboratory parameters. Although an active field, the overall role of biomarkers in sepsis diagnosis remains undefined (4, 38, 39). With improvements in RNA extraction methodologies, there has been a renewed focus toward cellular transcriptomic analysis in sepsis. Several groups have published similar studies (16, 17, 20–24, 29, 30, 33, 37, 40–44) with various biomarker configurations in clinical validation or development (12, 15–18, 21, 24, 45–49). Despite considerable advances, the field is still considered to be evolving and ‘significant work is needed to identify the optimal combinations of biomarkers that can augment diagnosis, treatment, and influence good patient outcomes’ (50).

We used a bioinformatics approach to identify candidate gene expression signatures across multiple cohorts of adult and pediatric patients and identified biomarker signatures centered around hub gene targets (25). Using this list of plausible biomarkers, we analyzed PBL mRNA in a new differential gene expression study and found high-functioning transcriptional signatures able to (i) identify severe systemic inflammation and (ii) differentiate SIRS from sepsis, in adult patients within the first 24 hours of ICU admission in a prospective, multi-center clinical study. Our work comprises an unparalleled, well-annotated clinical dataset, with a range of clinically relevant samples/measurements taken across the time course of the study. To our knowledge this is the first study to combine clearly defined and stratified disease groups based on clinical characteristics. We present temporal clinical and immune parameter alongside mRNA biomarker data, enabling identification of biomarkers useful for primary diagnosis, for prognosis and patient monitoring, which could be used in conjunction with other clinical measurements. We offer insights into the correlation between classical clinical measurements and biomarker expression and their possible relation to cellular/disease pathology, patient trajectory over the course of ICU stay and their relation to clinical outcomes.

We identified thirty-three high-performance, differentially expressed mRNA biomarkers between control and combined SIRS/Sepsis groups for severe inflammation and termed these ‘indicators of inflammation’ (I°I). We selected 19 entities for further detailed investigation, including CD177, FAM20A and OLAH. These exhibit highly similar expression patterns and most likely arise from a granulocyte population e.g. neutrophils. Providing external validation to our findings, several genes, such as CD177, ARG1 (arginase), MMP9, OLAH and ADM have been described previously as having important inflammatory roles in sepsis (16, 29, 41, 42, 44, 51–71). ARG1 in particular has been identified by other groups as a good biomarker for sepsis diagnosis (64), specifically associated with neutrophil activity (72) a component of which may be from a myeloid-derived suppressor cell (MDSC) phenotype (60). These have been postulated to promote immune-suppression during sepsis and may also serve the same function in SIRS due to surgery or trauma (73, 74), perhaps due to arginase suppression of T-cell function (55, 56, 63, 75). These may be molecular signatures referencing a neutrophil-to-lymphocyte ratio imbalance (NLR). NLR is a well-documented feature of many severe inflammatory conditions including sepsis (76), heart failure and other conditions (77), trauma (78, 79) and cardiac arrest (80–83) and is indicative of a poor outcome. Elevated neutrophil and reduced lymphocyte counts have been associated with poor outcomes in emergency medicine in general (84). The NLR response in sepsis is irrespective of age as it is also observed in neonatal sepsis (85, 86). We believe that our I°I biomarker profiles may be a reflection of this response, as some of the elevated biomarkers are cell-type specific for neutrophils e.g. CD177, MMP9 and ADM and appear inversely correlated with others which may be lymphocyte associated e.g. CD8b, LY9 and TCRα constant. The data presented here supports the premise that neutrophil recruitment/activation is a common feature of severe systemic inflammation and is not specific to sepsis. Schaack et al. reported OLAH, CD177, MMP8, RETN and HP as among the most upregulated genes in sepsis and separated them into two clusters of immune suppression and activation where some showed overlap in function (58). They concluded that in addition to a loss of monocyte and T-cell function and an increase in neutrophils and granulocyte numbers, many cells showed contradictory activation states.

Both infection-driven and sterile inflammation can lead to organ dysfunction through activation of similar innate immune pathways. A variety of Toll-like receptors may be activated via damage-associated molecular products. This may lead to development of neutrophil extracellular traps (75, 87). To date, protein-based approaches to distinguish between the infection-driven and sterile processes has been largely unsuccessful (87). Similarly, gene expression studies revealed significant congruence of signaling between these conditions, with up to 92% of genes showing change in the same direction (88). We used a novel ANN driven methodology in addition to parametric statistical methods, to counter the issues of a standard hypothesis-driven approach to find discriminative molecular biomarker patterns between sepsis and non-infective SIRS. Both Random Forest and ANN-based modeling are common tools used in biomarker discovery, due to their ability to classify nonlinear information with random sampling, while providing accurate predictions using a decision tree or mathematical function algorithm (28). Utilizing this approach enabled us to look beyond standard inflammasome markers, which have been shown to be similarly regulated in both infection and non-infection driven inflammation (88, 89). Our results pave the way for these delineated I°I signatures to be used to accurately identify severe inflammation at early stages of presentation. Arguably, clinical evaluation can differentiate between healthily controls and patients with SIRS or sepsis in our cohort of critically ill patients, however the clear difference in the clinical variables enabled us to find sensitive and specific biomarker panels. These will need to be further tested at earlier timepoints in apparently less tangible disease presentations.

Combinations of these I°I biomarkers were assessed for performance in delineating disease groups from healthy controls. A number of candidate I°I signature panels showed exceptional performance on our dataset, with a minimal configuration of FAM20A+OLAH showing good performance across all timepoints with an AUC of 0.9906-1.0. This test combination would clearly discriminate between healthy individuals and SIRS or sepsis and could be used to rapidly triage patients with suspected severe inflammation, either as a rule-in or rule-out tool. This performed equally well when analyzed on other datasets, both in adult and pediatric populations (16, 30, 31, 33, 34, 42, 43). This smaller set may be more clinically useful than a larger panel, from a test development rationale, as it may be cheaper and simpler to configure as multiplex qPCR or other assays. A larger 5-biomarker combination e.g. OLAH+FAM20A+ITGA7+MMP9+ADM may provide more resilience on broader, diverse, sample populations and provide better resolution through higher fold-change between groups in a composite panel score, but may be more challenging to configure.

Twenty select entities were differentially expressed between sepsis and SIRS, termed ‘SIRS or Sepsis’ (S°S) biomarkers. The best performing panel to differentiate sepsis from SIRS was CMTM5/CETP/PLA2G7/MIA/MPP3 using our dataset (AUC=0.9758). This 5-panel S°S signature achieved excellent diagnostic accuracy for abdominal and pulmonary sepsis versus SIRS in our cohort. Many of the individual candidate S°S biomarkers have been previously associated with sepsis. Cholesterol ester transfer protein (CETP), a lipid transfer glycoprotein, has been widely discussed as key target in the sepsis inflammatory response, particularly in sepsis caused by gram-negative infection (90–92). Upregulation of CETP has been associated with survival in sepsis (93, 94) and linked to modulation of HDL in resolving bacterial infections (90) and macrophage polarization (95). We have previously identified CMTM5 and ITGB3 as associates of the hub entity MYL9; key differentiators of Sepsis and SIRS with platelet activation function (25). PLA2G7 features in the Septicyte Lab Signature (16) as a downregulated entity. Additionally, down-regulation of the monocyte-associated ARHGEF10L has been previously associated with disease severity and ICU patient mortality (96, 97). MPP3 to our knowledge has not been previously associated with SIRS or sepsis.

We selected three cut-off values to delineate ranges over which the S°S biomarker assays could distinguish between individuals with SIRS and sepsis. The first, which provides excellent PPV (90-95%) for sepsis detection which could be used as a ‘rule in’ test to identify sepsis and begin antibiotic treatment. A second cut-off was selected which provides excellent (>98%) NPV for ruling out sepsis which could be used for ruling out bacterial infection and would prevent unnecessary antibiotic treatment to these patients. The third cut-off value lies in the middle where the groups overlap significantly and where sepsis may or may not be present. A test based on the use of ranges based on all three cut-off classifiers would be beneficial for patient care and could replace our current best guess protein biomarkers with improved accuracy.

One of the confounding differences between this study and other published biomarker discovery studies is the RNA extraction method used. We extracted mRNA from isolated PBLs using the erythrocyte cell lysis method, as opposed to use of PAXgene Blood RNA tubes, which are used in many other studies such as for discovery of the Septicyte Lab signature (16). Although there may be disadvantages with increased labor for processing of samples immediately at time of collection, mRNA extraction time is shorter and may produce differences in mRNA profiles. Differences in the two extraction methods and consequential changes in the gene expression profiles identified requires further investigation. It is hypothesized that our PBL extraction method may be useful in pulling down blood clots and extracellular traps excreted from neutrophils, revealing more sepsis-specific biomarkers associated with platelets and granulocytes, which may not appear as significantly differentially regulated in other studies.

The other significant difference between our study and of others is the timing of clinical and laboratory evaluation. Timing of sample collection in relation to the insult leading to organ dysfunction has been shown to be important when interpreting gene expression datasets (13). Our patients had a short prodrome and arrived at the ICU with predominantly community acquired infections or in the case of the SIRS group organ dysfunctions developed over a short time period. This important clinical characteristic, coupled with the significant, but comparable acute organ dysfunction in both groups may have helped to amplify the results observed in our cohorts. As approximately half of sepsis and overwhelming majority of post-cardiac arrest admissions to the ICU have a short lead-in time, our results could be clinically relevant for a large group of ICU patients (98). Furthermore, our observation that gene expression profiles changed from a SIRS-like pattern toward a sepsis-like pattern in the SIRS group around Day5, when clinical details indicated the presence of new, ICU-acquired infection, provides internal validation of the findings.

When validated in a prospective manner, these tools have the potential to significantly enhance the clinical diagnostic capabilities of the ICU and other lower dependency wards in sepsis. Despite the methodological differences between previous studies and ours, we successfully validated our biomarker signatures on multiple comparable gene expression datasets. We found that different combinations of the individual mRNA biomarkers can achieve good discriminatory power in these datasets. The reduced performance observed maybe in part due to study/platform technical variation. We also attempted to check if the previously published mRNA signature panels would perform as well as, or better than ours in the ANEMONES dataset. Interestingly, only two of the five previously published mRNA signatures were able to distinguish the sepsis and SIRS groups (12, 16, 22, 23, 37). This observation may be compounded by heterogeneity introduced through study protocol and/or technical differences and temporal endotype variation (99, 100). Both signatures were identified from studies comparing patients with sepsis to surgical patients with post-surgical systemic inflammation and patients with SIRS respectively, however these studies did not include a set of healthy controls which is a major difference to our study and may have a significant impact on the results (16, 37). Other more recent comparable studies have similarly lacked either one or other of SIRS or control groups (101, 102).

Our study has limitations. The patients in the SIRS group had a common unique clinical presentation ‘out-of-hospital cardiac arrest’, which may limit the generalizability of the findings of the SIRS features. However, in the temporal samples we have clearly observed a change in biomarker expression from the SIRS pattern to the sepsis pattern in patients who then developed ICU acquired infections. Similarly, in selected cases, patients presenting with sepsis started to exhibit SIRS pattern, where the clinical course involved cardiovascular events following the infectious episode. In addition, our S°S signature panels showed good performance in external datasets, where the SIRS groups had more varied clinical etiology. Our study was designed and completed before the Sepsis 3.0 definition was published in 2016, hence we continued to adhere to the terminology used in our protocol (4). Singer et al. described sepsis as dysregulated host response causing organ dysfunction secondary to infection (4). Their clinical criteria was presence of presumed or confirmed infection and a SOFA score of 2 or above, or an increase of the SOFA score of 2 or more, if it was not 0 before (4). Notably, all patients in the sepsis group would have been classified as sepsis using the Sepsis 3.0 definition as well, given that the lowest observed SOFA score was 7 in the sepsis groups. Given our gene expression data also demonstrating a dysregulated host response, secondary to infection, we are certain that our results remain current using the new sepsis definition. Although our sample size was relatively small, our study is readily comparable to other published datasets. In addition, unlike many other studies focusing solely on gene expression, we have cultivated a very rich clinical database and were able to track the clinical decision making throughout the patients ICU stay. While our results need independent validation in prospective new clinical cohorts, we have shown that our biomarker panels perform at least as well as previously published and patented biomarker signatures in historical datasets.

Overall, we revealed a unique two-tier strategy using two separate biomarker signatures to identify systemic inflammation and discriminate sepsis from non-infectious SIRS using the I°I and S°S signatures, respectively. We have discovered parsimonious sets of genes which in a two-tier model can differentiate between healthy controls and individuals with systemic inflammation with very high accuracy and are then able to discriminate between sepsis and SIRS. Firstly, the I°I signature can be utilized to identify Systemic Inflammation (patients with either sepsis or SIRS) followed by stratification of Sepsis (SIRS from infection) from SIRS (without infection) using the S°S Signature. Both signatures consist of 5 differentially expressed biomarkers, some of which many have been previously identified as sepsis-associated. We anticipate the I°I signature may be a useful triage test in multiple clinical settings, including ICU, lower dependency ward or community settings, to recognize ‘at risk’ patients. The S°S signature would be useful clinically for sepsis differential diagnosis, prediction of severity and patient outcome.

## Data availability statement

The datasets presented in this study can be found in online repositories. The names of the repository/repositories and accession number(s) can be found below: https://www.ncbi.nlm.nih.gov/,GSE236713.

## Ethics statement

The studies involving humans were approved by South Wales Research Ethics Committee Panel D, Reference number: 12/WA/0303; date of approval: 21/11/2012. The studies were conducted in accordance with the local legislation and institutional requirements and the Declaration of Helsinki. The participants, or their personal or legal representatives, as applicable, provided their written informed consent to participate in this study.

## Author contributions

TS: Conceptualization, Formal analysis, Funding acquisition, Investigation, Methodology, Project administration, Resources, Supervision, Visualization, Writing – original draft, Writing – review & editing. EF: Data curation, Formal analysis, Investigation, Methodology, Software, Validation, Visualization, Writing – review & editing. HG: Conceptualization, Data curation, Formal analysis, Investigation, Methodology, Software, Visualization, Writing – original draft, Writing – review & editing. TW: Investigation, Methodology, Project administration, Resources, Supervision, Writing – review & editing. TM: Formal analysis, Investigation, Methodology, Resources, Writing – review & editing. SS: Conceptualization, Formal analysis, Funding acquisition, Investigation, Methodology, Project administration, Resources, Supervision, Visualization, Writing – review & editing. DT: Data curation, Formal analysis, Investigation, Software, Visualization, Writing – review & editing. JH: Conceptualization, Formal analysis, Funding acquisition, Investigation, Methodology, Project administration, Resources, Supervision, Visualization, Writing – review & editing. GB: Conceptualization, Formal analysis, Funding acquisition, Investigation, Methodology, Project administration, Resources, Software, Supervision, Visualization, Writing – review & editing. KK: Conceptualization, Data curation, Formal analysis, Funding acquisition, Investigation, Methodology, Project administration, Resources, Software, Supervision, Visualization, Writing – original draft, Writing – review & editing.
