# Supplementary figures and images for: The ‘analysis of gene expression and biomarkers for point-of-care decision support in Sepsis‘ study; temporal clinical parameter analysis and validation of early diagnostic biomarker signatures for severe inflammation andsepsis-SIRS discrimination
Source: Front Immunol. 2024 Jan 25;14:1308530. doi: 10.3389/fimmu.2023.1308530 (PMC10850284; doi:10.3389/fimmu.2023.1308530)

## Slide 1
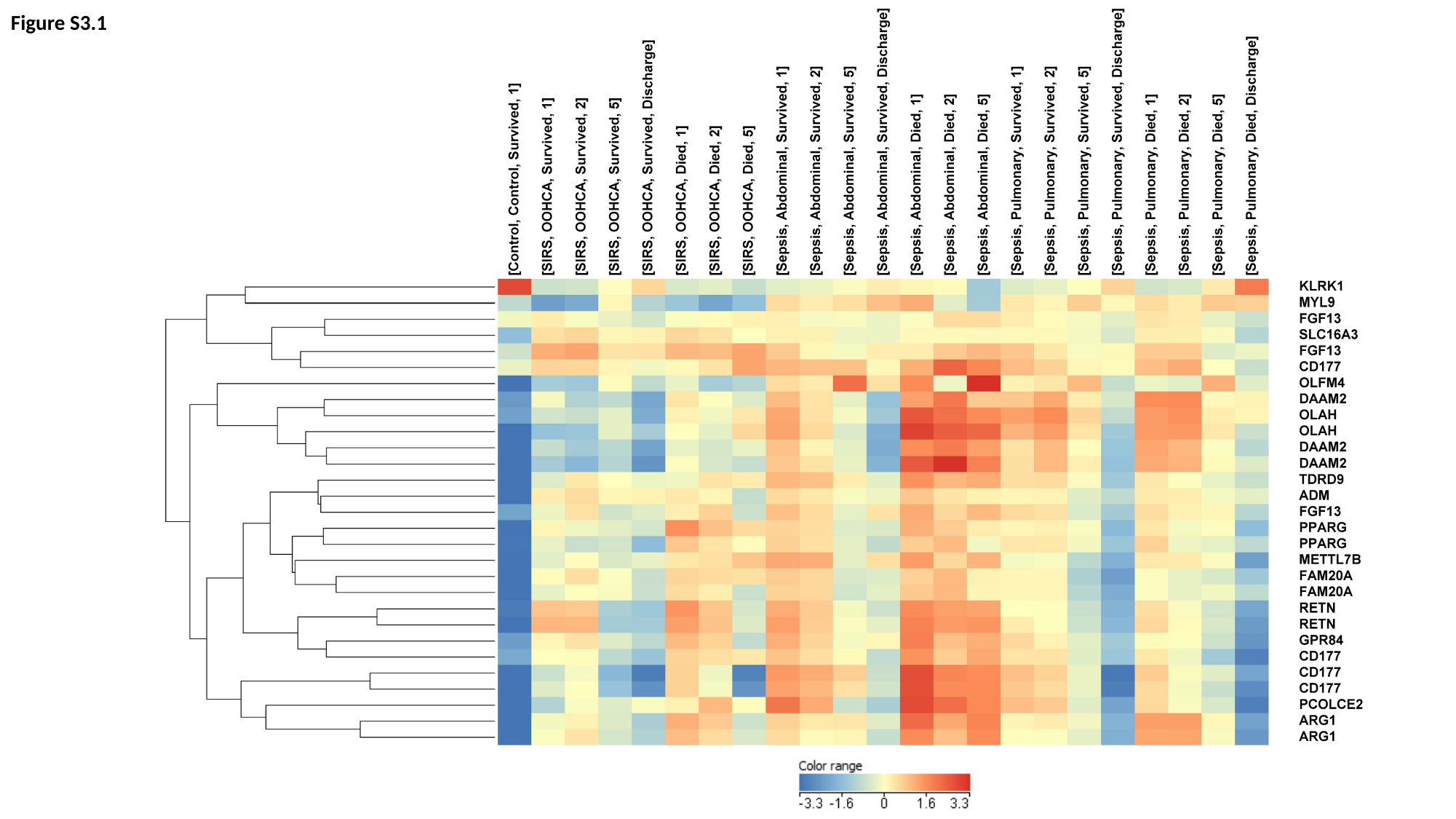

Figure S3.1

Supplement: Supplementary file 1 [file Presentation_1.pptx]

## Slide 1
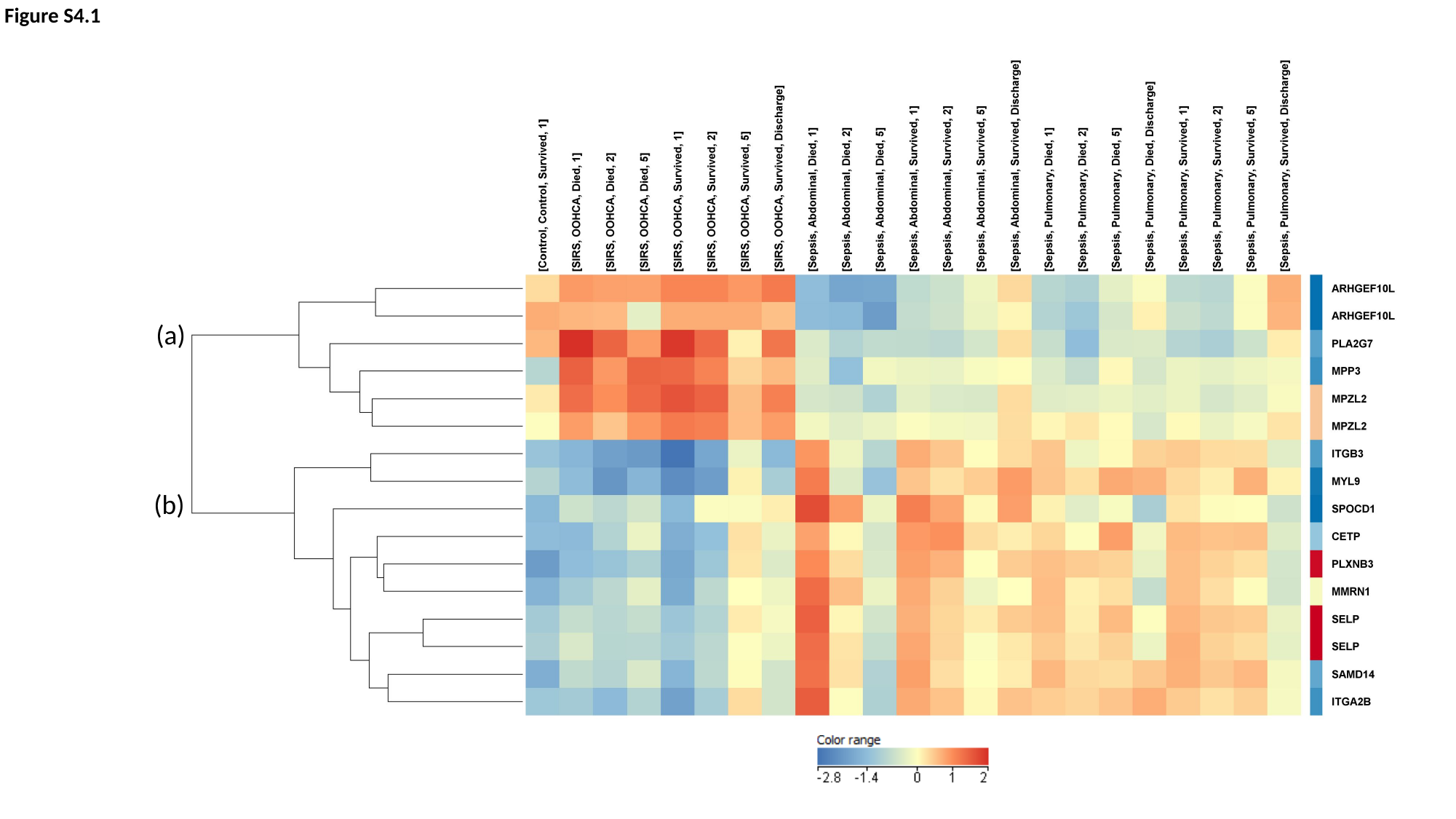

Figure S4.1
(a)
(b)

Supplement: Supplementary file 2 [file Presentation_2.pptx]
